# Pyroptosis-Related Signature as Potential Biomarkers for Predicting Prognosis and Therapy Response in Colorectal Cancer Patients

**DOI:** 10.3389/fgene.2022.925338

**Published:** 2022-07-22

**Authors:** Zhiyong Li, Yang Liu, Baiqiang Lin, Wei Yan, Huijie Yi, Haoran Wang, Yunwei Wei

**Affiliations:** ^1^ Department of Oncological and Endoscopic Surgery, The First Affiliated Hospital of Harbin Medical University, Harbin, China; ^2^ Translational Medicine Research and Cooperation Center of Northern China, Heilongjiang Academy of Medical Sciences, Harbin, China; ^3^ Pancreatic and Gastrointestinal Surgery Division, HwaMei Hospital, University of Chinese Academy of Science, Ningbo, China; ^4^ Peking University School of Nursing, Beijing, China; ^5^ Respiratory and Critical Care Medicine, Peking University People’s Hospital, Beijing, China

**Keywords:** colorectal cancer, pyroptosis, prognostic, genetic alteration, immune cell infiltration, single-cell sequencing

## Abstract

**Background:** Abnormal mucosal inflammation is a critical risk factor for pathogenesis and progression of colorectal cancer (CRC). As a type of proinflammatory death, pyroptosis can recast a suitable microenvironment to promote tumor growth. However, the potential role of pyroptosis in CRC remains unclear.

**Methods:** A total of 38 pyroptosis-related gene (PRG) expression profiles and clinical information were collected from multiple public datasets. Bioinformatics methods were used to analyze the clinical significance, functional status, immune infiltration, genomic alteration, and drug sensitivity in different subgroups. Whole-genome microarray analysis was performed to analyze the regulation of gut microbiota on the expression of PRGs.

**Results:** Two distinct molecular subtypes were identified and suggested that multilayer PRG alterations were associated with patient clinicopathological features, prognosis, and tumor microenvironment (TME) infiltrating characteristics. Furthermore, we obtained eight PRG signatures by applying differential expression analysis and univariate Cox and Lasso regression analyses. A risk prognosis model was constructed for predicting overall survival (OS) and recurrence-free survival (RFS) based on the PRG signature. There were significant differences in clinical characteristics, 22 immune cells, and immune functions between the high- and low-risk groups. In addition, the PRG signature was significantly associated with the microsatellite instability (MSI), tumor mutation burden (TMB), cancer stem cell (CSC) index, immunotherapeutic characteristics, and chemotherapeutic drug sensitivity. Moreover, the *in vitro* experiments had shown that *Fusobacterium nucleatum* (*F.n*) could affect the CASP6 expression, which was associated with the chemoresistance to 5-fluorouracil (5-Fu) in CRC.

**Conclusion:** Our findings provided a foundation for future research targeting pyroptosis and a new insight into the prognosis and immune cell infiltration of CRC, and they suggested that *F.n* might influence CRC progression through pyroptosis.

## Introduction

CRC is one of the most common malignant tumors and the second leading cause of cancer-related death worldwide (Cartwright, 2012; Siegel et al., 2013). The morbidity rate of CRC remains on the rise: currently, nearly 1.8 million people have been diagnosed, and more than 900,000 people die each year from CRC (Sung et al., 2021). Despite recent advances in surgical and multimodal therapies, the 5-year survival rate remains poor for patients with postoperative recurrence and advanced CRC patients (Zhang L et al., 2019; Ganesh et al., 2019). The TMN stage system has been widely used for clinical practice to predict patient prognosis and therapy decision-making. Dishearteningly, CRC has a complex status due to its heterogeneity; for instance, abnormalities in the TME may lead to widespread tumor heterogeneity and further results in significant heterogeneity with regard to the response to therapy and heterogeneity in their clinical outcomes. Therefore, the development of an effective gene signature to indicate prognosis and to guide clinical treatment in CRC patients, especially with regard to immunotherapy and chemotherapy, is needed. Notably, prolonged inflammation is one of the characteristics of CRC, and a key driver of CRC development is the inflammatory response pathway (Lasry et al., 2016; Keum and Giovannucci, 2019).

Pyroptosis is an exceptional form of inflammatory cell death compared to other types of programmed cell death (Fang et al., 2020), and plays an important role in antagonizing infection and endogenous danger signals. Pyroptosis can be induced by activation of the executors, gasdermin E (GSDME), or gasdermin D (GSDMD), which results in the cleavage of their N-terminal fragments (GSDME-N or GSDMD-N, respectively) (Shi et al., 2015; Wang et al., 2017; Feng et al., 2018). Many studies have revealed that pyroptosis plays a crucial role in the pathogenesis and progression of CRC(Wang et al., 2019; Ruan et al., 2020; Tan et al., 2020). CRC is characterized by inherent biological invasiveness and specific radiological and chemical resistance that result in high recurrence rates and progression. GSDMD expression is decreased in CRC cells compared to that in adjacent normal cells, and low GSDMD expression is associated with a worse CRC prognosis. Moreover, lipopolysaccharide (LPS) induces pyroptosis of CRC cells to improve chemosensitivity in response to oxaliplatin via promoting GSDMD-N membrane translocation (Wu et al., 2020). Recently, Yu et al. (2019)have reported that JNK is involved in lobaplatin-induced CRC cell pyroptosis by activating the caspase 3/GSDME signaling pathway. Furthermore, the TME, which is a complex system composed of tumor cells, lymphocytes, cancer-associated fibroblasts, and other cells, can affect cancer development and progression (Pottier et al., 2015; Nicholas et al., 2016). There is increasing evidence that indicates cross-talk between pyroptosis and the TME mediates tumor development and progression (Runa et al., 2017; Orning et al., 2019; Erkes et al., 2020). Pyroptosis creates a tumor-suppressive microenvironment by releasing inflammatory factors; nevertheless, it can also impair the body’s immune response to tumor cells and accelerate tumor growth in different cancers (Zaki et al., 2010; Ma et al., 2016). However, the association between pyroptosis and TME, especially with regard to immune cell infiltration and prognosis of CRC, remains unclear.

Here, several large public databases were used to perform comprehensive bioinformatics analysis of PRG in CRC, including genetic alterations, expression, prognosis, immune cell infiltration, and functional analysis. First, 585 CRC patients from GSE39582 were clustered according to PRG expression levels and found that PRG alterations were related to prognosis and immune cell infiltration. Furthermore, a PRG signature model was constructed by using LASSO-Cox method based on the GEO and TCGA datasets. This PRG signature showed considerable performance, which was confirmed by internal and external validation. In addition, This PRG signature model was able to predict prognosis, immune cell infiltration, immunotherapy, and chemotherapy response. On this basis, we found that there could be an association between pyroptosis, *F.n,* and chemoresistance to 5-Fu in CRC. To validate this association, human CRC cells were incubated with or without *F.n in vitro*. We initially determined that the expression of CASP6 could be downregulated by *F.n* and further induces chemoresistance of CRC cells to 5-Fu. Our evidence suggested that the expression levels of CASP6 might serve as promising prognosis biomarkers and therapeutic targets, and also revealed the potential association between gut microbiota and pyroptosis in CRC.

## Materials and Methods

### Data Source Preparation and Preprocessing

The overall flow diagram is shown in Supplementary Figure S1. Three GEO CRC cohorts (ID: GSE39582, GSE17536, and GSE90944) were downloaded from the Gene Expression Omnibus (GEO) database (https://www.ncbi.nlm.nih.gov/geo/), and the relevant prognostic and clinicopathological data are publicly available in the GEO database. The RNA sequencing (FPKM value) data, which were transformed into transcripts per kilobase million (TPM), including 568 tumor samples and 44 normal samples, as well as corresponding clinical information regarding CRC, were downloaded from The Cancer Genome Atlas (TCGA, https://portal.gdc.cancer.gov/) data portal on 08 November 2021. Four datasets were combined, and batch effects were eliminated by using the “ComBat” algorithm of the “SVA” package. The data acquisition of the list of the immune genes was downloaded from the ImmPort database (https://www.immport.org). The 38 PRGs were identified from the published literature (Broz et al., 2020; Tan et al., 2020; Shao et al., 2021; Song et al., 2021; Ye et al., 2021), which are shown in Supplementary Table S1.

### Consensus Clustering Analysis

According to the expressions of 38 PRGs, the R package “ConsensusClusterPlus” was used to clarify patients into distinct molecular subtypes by the cophenetic correlation coefficient (k-means method) (Liu et al., 2022a). Repetitions were performed 1,000 times to guarantee the stability of distinct classification. Effective dimensionality reduction, model recognition, and grouping visualization of high-dimensional data for the different clustering subtypes were performed by principal component analysis (PCA). The relationships between distinct subtypes and clinicopathological characteristics were performed to examine the clinical value. In addition, we assessed the differences in OS and RFS in distinct subtypes using Kaplan–Meier survival analysis by the “survival” and “survminer” R packages.

### Construction and Validation of the PRG Signature

To evaluate the correlations between each PRGs and survival status, we screened the prognosis of PRGs by univariate Cox regression analysis in GSE39582 cohort. We then analyzed LASSO Cox regression to develop the prognostic model by using the “glmnet” R package. The risk score was calculated according to the following: Risk score = ∑Expi * βi (Expi, each PRG signature expression; βi, each PRG signature coefficient). For the internal validation studies, the GSE39582 cohorts were divided into low- and high-risk groups based on the median risk score. Receiver operating characteristic (ROC) analysis was used to assess the accuracy of PRG signature in the training dataset and testing dataset by using the “timeROC” R package. Moreover, the OS and RFS were compared between the low- and high-risk groups via Kaplan-Meier analysis by the “survival” and “survminer” R packages. Furthermore, PCA was performed by using the “ggplot2” R package. For the external validation studies, the patients in the GSE17536 and TCGA cohorts were also divided into low- and high-risk groups to validate the risk model based on the median risk score from the GSE39582 cohort.

### Functional Enrichment Analysis

GSVA enrichment analysis was performed in heatmap by using the “GSVA” R packages, and “c2.cp.kegg.v6.2.symbols” from the MSigDB database to carry out GSVA analysis. FDR <0.05 was considered to indicate statistical significance in distinct subtypes based on the “limma” package. In addition, the differentially expressed genes (DEGs) between the low- and high-risk groups were identified by using the “limma” R package according to specific criteria (fold-change of 1.5 and FDR <0.05). The Gene Ontology (GO) and Kyoto Encyclopedia of Genes and Genomes (KEGG) analyses were performed by applying the “clusterProfiler” R package based on these DEGs.

### Stratification Analysis and Independent Prognostic Analysis of the PRG Signature

We first extracted the clinical features of patients in the GEO and TCGA cohort. To determine the predictive ability of the PRG signature, we further performed a stratified analysis based on these clinical characteristics in low- and high-risk groups. To explore whether risk scores were independent of other clinical features (age, gender, and stage), univariate and multivariable Cox regression models were employed for the analysis in training and testing sets.

### TME Cell Infiltration

The immune and stromal scores of TME were evaluated by the ESTIMATE algorithm (Liu et al., 2021a; Liu et al., 2021b; Liu et al., 2022a; Liu et al., 2022b), and the infiltration of 22 human immune cell subsets was calculated by the CIBERSORT algorithm for each patient (Newman et al., 2015). The correlations between the distinct groups and immune checkpoints also were analyzed. The infiltrative fractions of 22 immune cell types in different groups were visualized by using the “vioplot” R package. The ssGSEA was performed to evaluate the scores of infiltrating immune cells and the activity of immune-related pathways by using the “GSVA” R package. The correlation between PRG signature and immune infiltration was investigated by using the Tumor IMmune Estimation Resource (TIMER 2.0, https://cistrome.shinyapps.io/timer/).

### Construction of a Nomogram for Prediction

Based on the outcome of the independent prognosis analysis (risk score and other clinical predictors), the nomogram prediction model was set up by using the “rms” R package for the 1-, 3-, and 5-year OS and RFS. The calibration and accuracy of the nomogram were verified by the calibration plot (bootstrap methods with 1,000 replicates).

### Mutation, Tumor Mutation Burden, and Microsatellite Instability Analysis

The mutation frequency and oncoplot/waterfall plot of 38 PRGs were drawn from the TCGA-COAD/READ database by using the “maftools” R package. The location of CNV alteration of 38 PRGs on 23 chromosomes from the TCGA database was generated by using the “RCircos” R package. The correlation between PRG signature expression with TMB and MSI score was calculated by using spearman’s correlation analysis (*p* < 0.05 was considered statistically significant).

### Bacterial Strains and Cell Lines

*Fusobacterium nucleatum* (*F.n*) strain ATCC 25586 was purchased from American Type Culture Collection (ATCC) and was cultured in brain heart infusion (BHI) broth at 37°C under anaerobic conditions. The colon cancer cell lines HCT116 and HT29 were obtained from ATCC and grown in McCoy’s 5A (Gibco, United States). All cell culture medium was supplemented with 10% FBS (Gibco) and 1% penicillin and streptomycin (Beyotime, China) and cultured at 37°C in a humidified 5% CO2 atmosphere. Cells were exposed to *F.n* with a multiplicity of infection (MOI) of 100 (medium without antibiotics) (Yu et al., 2017; Zhang S et al., 2019).

### Real-Time PCR and Plasmid Transfection

Total RNA was extracted from normal and tumor tissues by TRIzol reagent (Invitrogen, Carlsbad, CA, United States). The total RNA was performed to synthesize complementary DNA (cDNA) by using the PrimeScript RT reagent Kit (TaKaRa, Japan). The cDNAs were subjected to SYBR Green assays (TaKaRa) based RT-qPCR on a CFX-96 instrument (Bio-Rad Laboratories). The Ct values obtained from different samples were compared using the 2^−ΔΔCT^ method and GAPDH served as internal reference genes. The primers used in real-time PCR assays were listed in Supplementary Table S2. The full length of CASP6 was amplified and inserted into a pcDNA3.1 vector (Invitrogen) to construct the CASP6 plasmid. Related oligonucleotides were transfected into colon cancer cell lines by using Lipofectamine 3,000 (Invitrogen, United States).

### Drug Susceptibility Analysis, Cell Proliferation Assay, and Drug Cytotoxicity Assay

The semi-inhibitory concentration (IC50) values of chemotherapeutic drugs usually used to treat CRC were calculated by using the “pRRophetic” R package. Spearman’s correlation analysis was performed to calculate the correlation between PRG signature expression and chemotherapeutics IC50 values (statistical significance was set at *p* < 0.05). Cell Counting Kit-8 (CCK8; YEASEN, China) was performed to assay the percentage of viable cells based on different treatment conditions. First, cells were seeded in 96-well plates with 100 μl culture medium. Second, the 10 μl of CCK-8 solution was added to each well at specific time points and incubated at 37°C for 4 h. The reaction product was measured according to the manufacturer’s protocol.

### Statistical Analysis

All statistical analyses were performed by R software (v4.1.0), GraphPad Prism (version 9.0), and SPSS software (23.0). *p* < 0.05 was considered statistically significant for all analysis results.

## Results

### Overview of the Genetic Alterations and Expressions of PRGs in CRC

**FIGURE 1:**
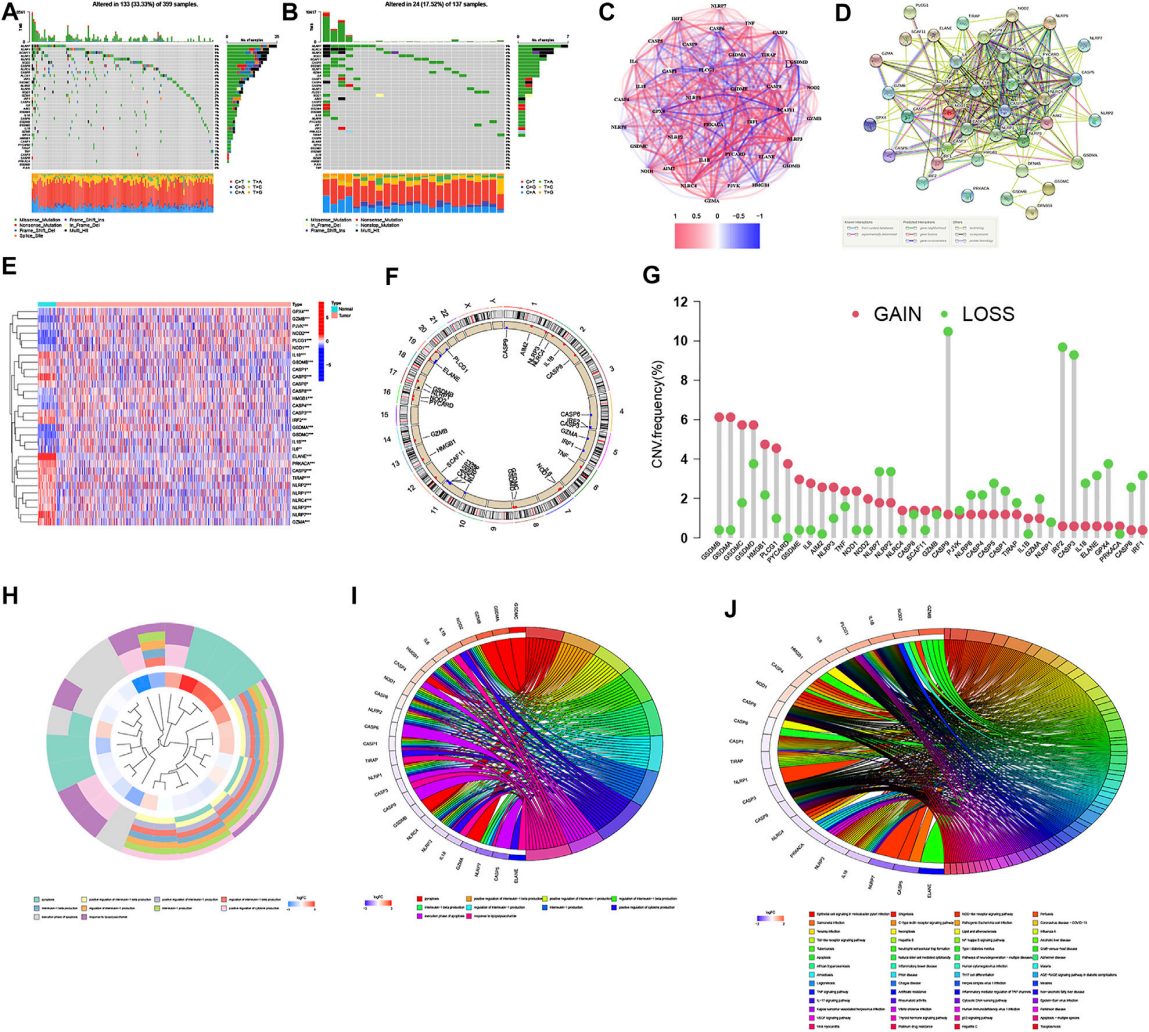
Landscape of the genetic alterations, expressions, and functions of PRGs in CRC. **(A,B)** Mutation frequencies of 38 PRGs in 399 and 137 patients with COAD and READ based on the TCGA cohort, respectively. **(C)** Correlation network of PRGs (the depth of the colors reflects the strength of the relevance, red line: positive correlation; blue line: negative correlation). **(D)** PPI network showing the interactions of PRGs (the minimum required interaction score was 0.4). **(E)** Heatmap of the PRGs between the normal and the tumor tissues (blue: low expression level; red: high expression level). *p < 0.05; **p < 0.01; and ***p < 0.001. **(F,G)** Locations of CNV alterations in PRGs on 23 chromosomes and the frequencies of CNV gain, loss, and non-CNV among PRGs, respectively. **(H–J)** Functional enrichment analysis of the differential expression levels of 30 PRGs ((H): heatmap clustering functional analysis; **(I)** enriched item in gene ontology analysis; **(J)** enriched item in Kyoto Encyclopedia of Genes and Genomes analysis).

Our study integrated 1,209 patients from three eligible CRC cohorts (TCGA-COAD/READ, GSE39582, and GSE17536) to fully investigate the genetic alterations, expression patterns, and prognostic values of 38 PRGs involved in tumorigenesis and development. We first explored the somatic mutation patterns of 38 PRGs in the COAD and READ cohorts. As shown in Figure 1A, 133 (33.33%) had mutations of these PRGs in 399 COAD samples. Among them, NLRP7 had the highest mutation frequency (6%), while five PRGs (CASP3, CASP6, PRKACA, GSDME, and PJVK) did not have any mutations. Meanwhile, the READ cohort had a lower PRG mutation frequency (17.52%, 24/137), and NLRP7 also had the highest mutation frequency (5%) (Figure 1B). In addition, we also constructed the correlation network and a protein–protein interaction (PPI) analysis to detect the interactions of these PRGs (Figures 1C,D). The mRNA expression levels were then performed to compare CRC and normal tissues and found that a total of 30 PRGs were either upregulated or downregulated in CRC patients (heatmap: Figure 1E, boxplot: Supplementary Figure S2A). Furthermore, we explored somatic copy number alterations in these PRGs. The copy number variation (CNV) alterations on their respective chromosomes as shown in Figure 1F and found prevalent copy number alterations in all 38 PRGs (Figure 1G). Notably, we discovered that the expression levels of most PRGs were positively correlated with CNV alteration, such as CASP1, CASP3, CASP6, GZMA, GSDMB, and NLRP1 were expressed at lower levels in CRC samples compared with normal samples, while GSDMC and CASP8 were significantly elevated in CRC samples. These results indicated that CNV might regulate the PRG expression in CRC. We further performed to explore the cluster functional analysis based on the differential expression levels of 30 PRGs. Heatmap clustering revealed that these genes were significantly involved in the positive regulation of cytokine production, interleukin-1 production, regulation of inflammatory response, pyroptosis, and execution phase of apoptosis (Figure 1H). GO enrichment analysis and KEGG pathway analysis were then performed to identify which PRGs are significantly associated with these functions. We found that a total of 25 of the 30 PRGs were mainly correlated with immunity in GO analysis (Figure 1I). Moreover, KEGG pathway analysis suggested that 22 PRGs were mainly enriched in immune and cancer-related pathways, including the *Helicobacter pylori* and salmonella infection, Toll-like and NOD-like receptor signaling pathway, VEGF and TNF signaling pathway, drug resistance and apoptosis (Figure 1J). We also divided the CRC patients into a low-stage group (stages I + II) and a high-stage group (stages III + IV) based on their clinical characteristics. A total of 14 of the 38 PRGs were identified as significantly differentially expressed in the two groups (Supplementary Figure S1B). Notably, the expression levels of 5 PRGs (CASP1, CASP3, CASP6, GSDMB, and GZMA) were lower in both CRC tissues and the high-stage group, suggesting that their potential function as tumor suppressors in CRC tumorigenesis and development.

### Identification of a Pyroptosis-Related Subtype in CRC

**FIGURE 2:**
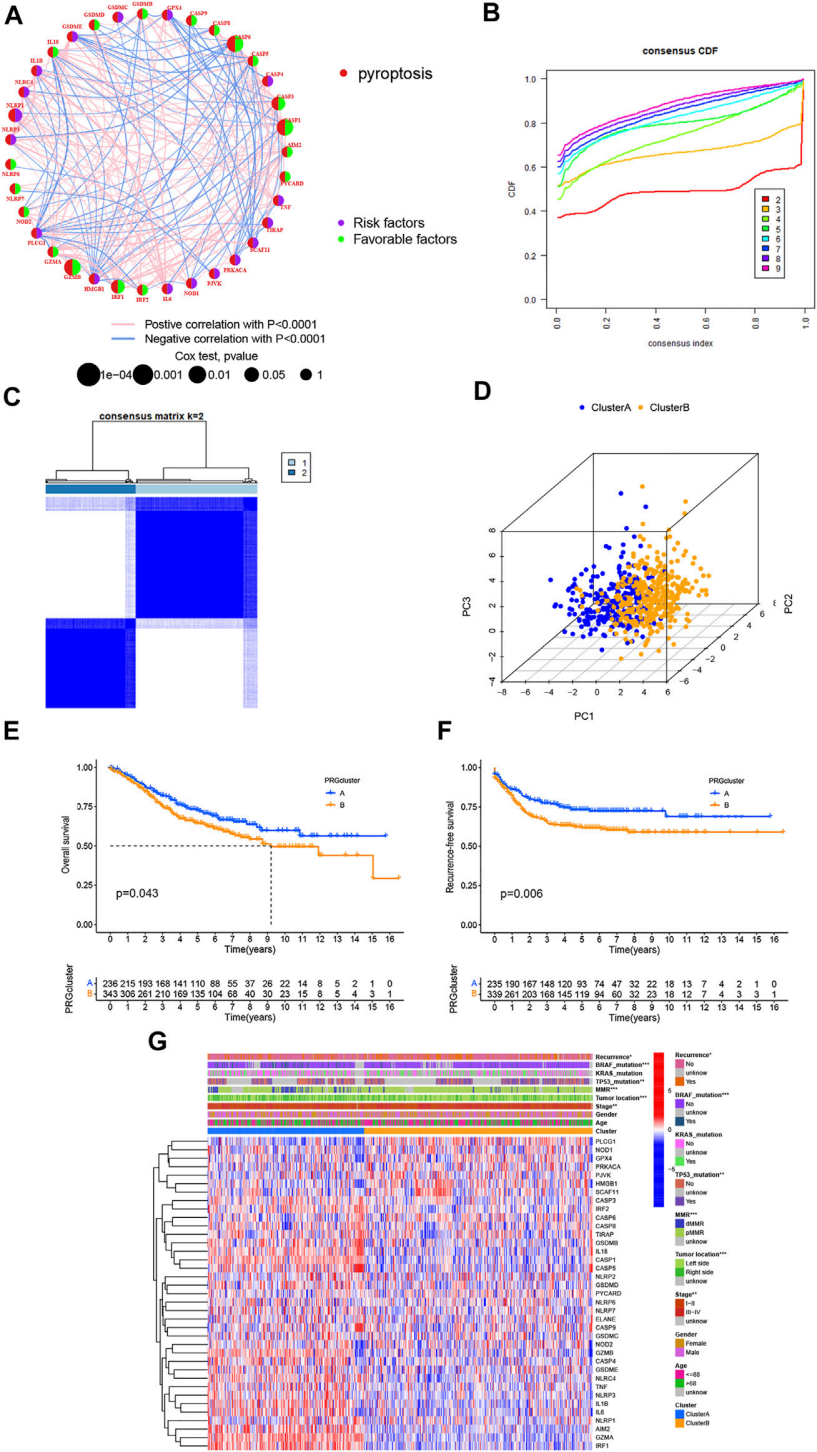
Two distinct subtypes of CRC are related by 37 PRGs based on consistent clustering. **(A)** Univariate cox regression analysis of OS for each PRGs in GSE39582 cohorts. **(B,C)** Consensus matrix heatmap defining two clusters (k = 2) and their correlation area. **(D)** PCA showed a remarkable difference in transcriptomes between the two subtypes, respectively. **(E,F)** OS and RFS curves showing 37 PRGs for the two distinct subtypes, respectively. **(G)** Differences in clinicopathological and biological characteristics of two distinct subtypes. *p < 0.05; **p < 0.01; and ***p < 0.001.

GSE39582 was selected as the exploration cohort for further analysis because it was the largest, most comprehensive, and most complete data series among the three datasets. Except for the GSDMA, other 37 PRGs were extracted and the prognostic values in CRC patients were revealed by using univariate Cox regression (Supplementary Table S3: OS, Supplementary Table S4: RFS, respectively). In addition, a pyroptosis network was constructed to visualize the landscape of PRG interactions, connections, and their prognostic values (Figure 2A). We further performed consensus clustering analysis to identify different pyroptosis-related subtypes based on 37 PRGs. Results showed that the patients were separated into two different subtypes according to the optimal clustering stability value (k = 2), including 236 cases in cluster A and 343 cases in cluster B (Figures 2B,C). Moreover, PCA demonstrated discernible dimensions between Cluster A and Cluster B (Figure 2D). In addition, The Kaplan–Meier curves showed that the OS and RFS of Cluster B for CRC patients were worse than that of Cluster A (*p* = 0.043 and *p* = 0.006, respectively) (Figures 2E,F). Moreover, the expression profile of 37 PRGs and their association with clinical characteristics was presented in a heatmap. As shown in Figure 2G, Cluster A was significantly related to lower TNM stage (*p* < 0.01), right-sided CRC (*p* < 0.001), higher deficient mismatch repair (dMMR) (*p* < 0.001), without TP53 (*p* < 0.01) and BRAF mutations (*p* < 0.001), and lower recurrence risk (*p* < 0.05) compared to those in Cluster B. Given the significant influence of PRG expression on the function of immunity (Figures 1I,J), we next investigated the distinct characteristics of the TME infiltration between Cluster A and Cluster B.

### Two Pyroptosis-Related Subtypes Associated With Distinct TME Infiltration

**FIGURE 3:**
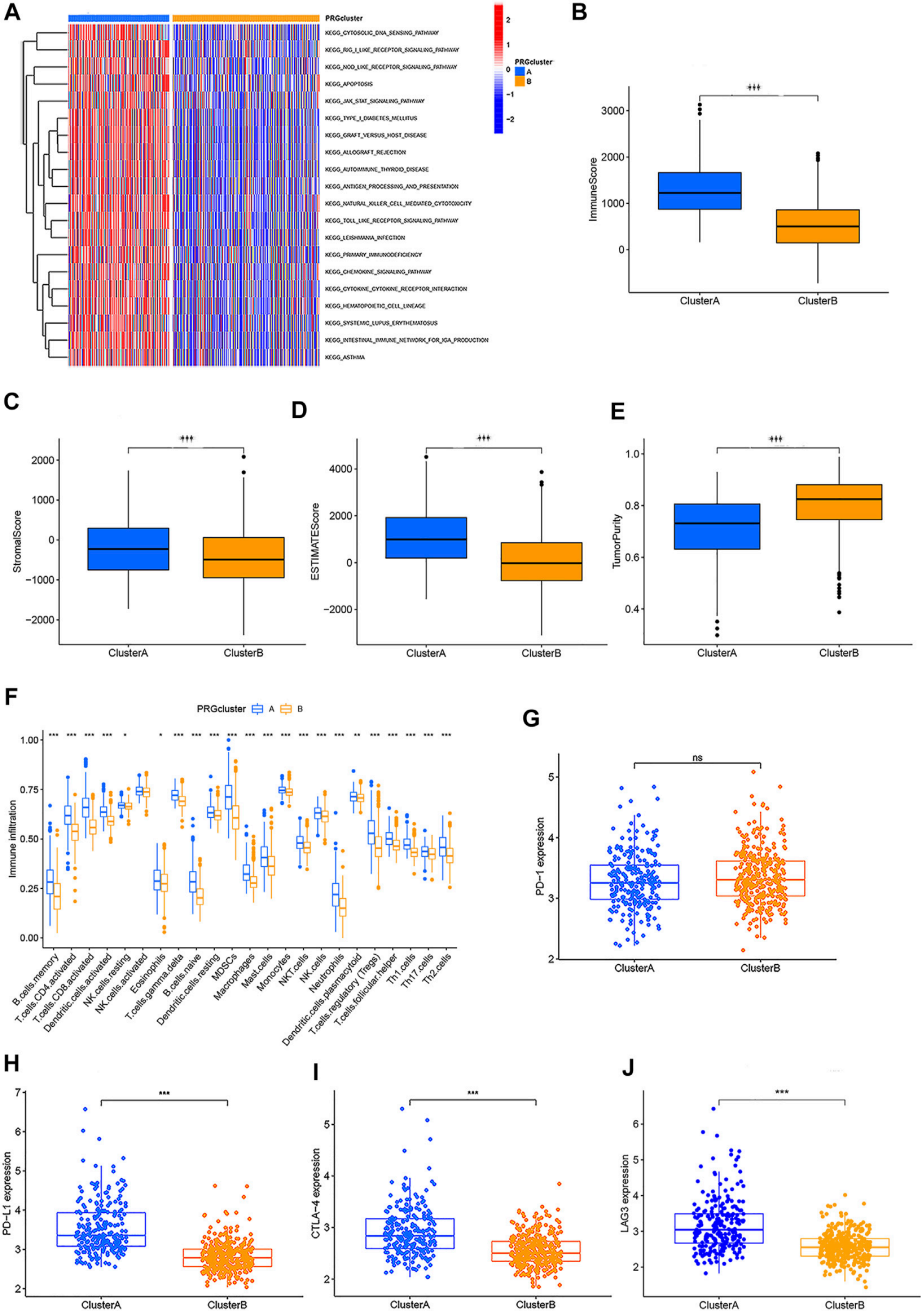
Two different CRC subtypes showed diverse tumor immune cell microenvironments. **(A)** Biological processes analyzed by GSVA which showed the active biological pathways in distinct subtypes. **(B–E)** Expression of the TME score and tumor purity in distinct subtypes. **(F)** Abundance of 23 TME infiltrating cells between the two CRC subtypes. **(G–J)** Expression levels of PD-1, PD-L1, LAG3, and CTLA-4 in the two CRC subtypes. *p < 0.05; **p < 0.01; and ***p < 0.001.

GSVA enrichment analysis was performed to identify the differences in biological behavior between these two subtypes. As shown in Figure 3A, Cluster A was significantly enriched in immune fully-activated pathways, including natural killer cell-mediated cytotoxicity, the activated JAK-STAT signaling pathway, antigen processing and presentation, cytokine receptor interaction, chemokine signaling pathway activation, intestinal immune network, RIG-I-like, NOD-like, and Toll-like receptor signaling pathways. To further investigate the correlation with TME infiltration in distinct subtypes, we analyzed the differences in terms of ImmuneScore, StromalScore, and EstimateScore between Cluster A and Cluster B. Our results revealed that Cluster B had a lower ImmuneScore, StromalScore, and EstimateScore than Cluster A, which indicates that Cluster B had a higher tumor purity than Cluster A (*p* < 0.001) (Figures 3B–E). According to the CIBERSORT algorithm, we observed that except for the NK cells activated, other immune cells were all poorly activated in Cluster B (Figure 3F). Similarly, we then performed to analyze the expression of four important immune-oncology targets (PD-1, PD-L1, LAG3, and CTLA-4) between Cluster A and Cluster B. As shown in Figures 3G–J, the expression levels of PD-L1, LAG3, and CTLA-4 were higher in Cluster A than in Cluster B (*p* < 0.001). Taken together, our results indicated that all PRGs were involved in shaping the TME infiltration, and represented different prognostic characteristics in CRC patients.

### PRG Signature Construction and Validation of a Risk Model in CRC

**FIGURE 4:**
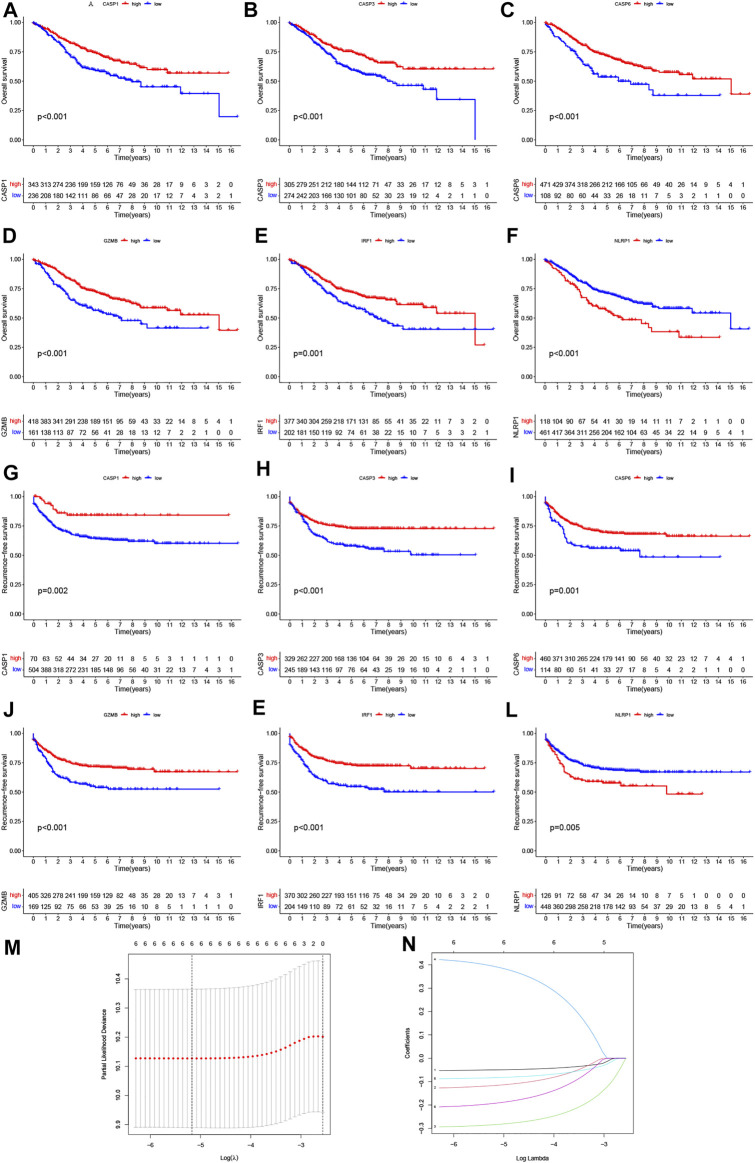
Prognostic value of six PRGs in CRC. The Kaplan–Meier curves of CASP1 **(A,G)**, CASP3 **(B,H)**, CASP6 **(C,I)**, GZMB **(D,J)**, IRF1 **(E,K)**, and NLRP1**(F, L)** for the low- and high-expression groups with the cut-off value 2.732 for OS and 2.281 for RFS in GSE39582 cohorts. **(M)** Cross-validation for tuning the parameter selection in the LASSO regression. **(N)** LASSO regression of the OS-related PRGs.

**FIGURE 5:**
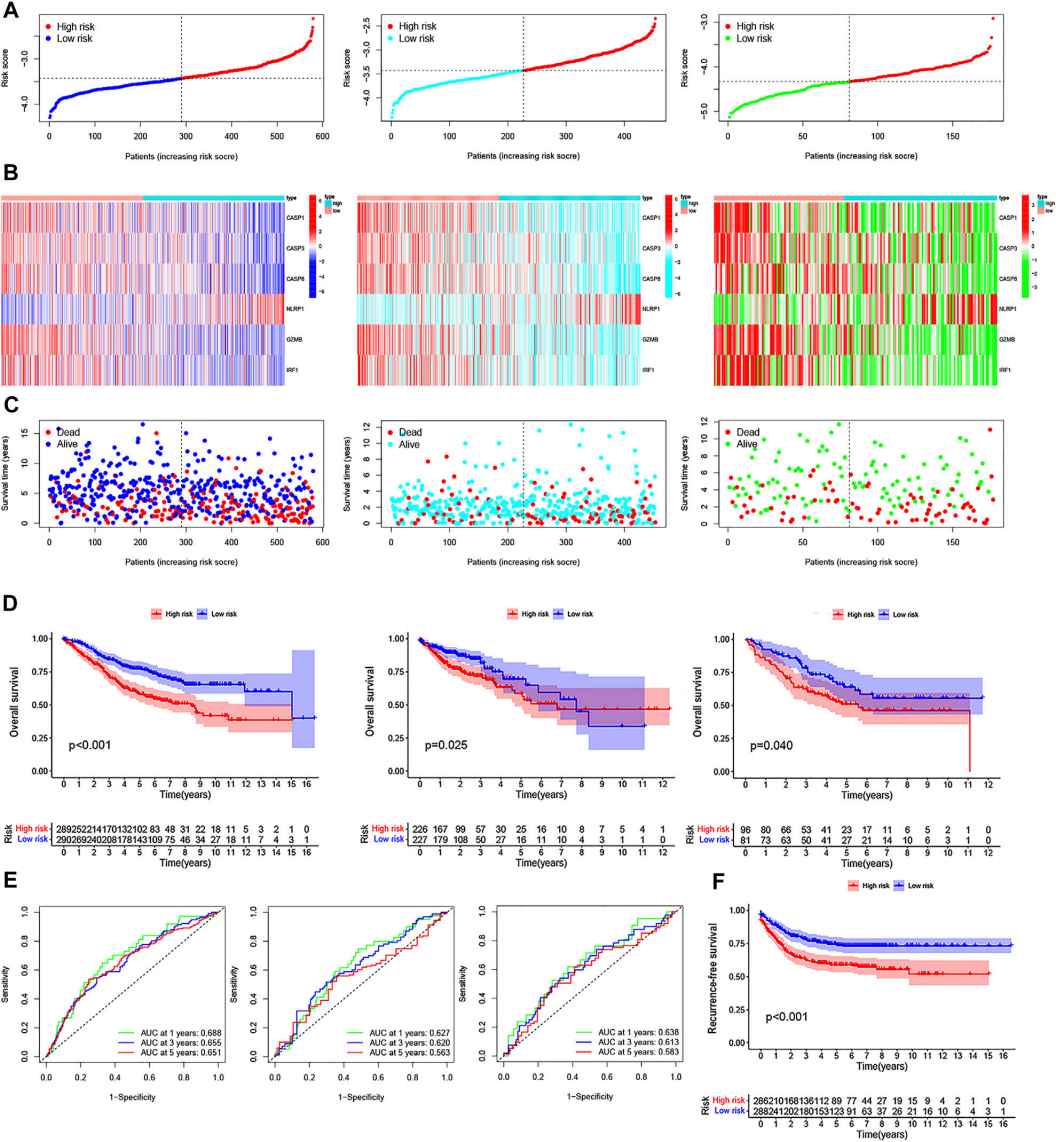
Construction of the PRG signature to predict patient survival. **(A–C)** Distribution of the risk score, survival time and survival status, and the heatmap of the six PRGs signatures in GSE39582, TCGA, and GSE17536 cohorts between the high- and low-risk groups, respectively. **(D)** Kaplan–Meier survival curves of the OS for patients in GSE39582, TCGA, and GSE17536 cohorts between the high- and low-risk groups, respectively. **(E)** 1-, 3-, and 5-year ROC curves of the PRG signature for the OS prediction in GSE39582, TCGA, and GSE17536 cohorts, respectively. **(F)** Kaplan–Meier survival curves of the relative RFS between the high- and low-risk groups in the GSE39582 cohort.

The Kaplan–Meier analysis was performed to determine whether these pyroptosis-related prognostic genes were associated with OS and RFS of CRC patients. As shown in Figures 4A–L, Kaplan–Meier curves showed that all the 6 PRGs were survival-associated with both OS and RFSin the high-/low-expression groups. To construct a prognostic PRG signature, we then performed a LASSO penalized Cox regression analysis to construct a six-PRG signature model in the exploration cohort (Figures 4M, N). Meanwhile, the patient samples were randomly divided into the training dataset (N = 291) and the test dataset (N = 288) at a ratio of 1:1. Therefore, the risk score for each patient in the training and testing dataset were calculated based on the risk formula: risk score = CASP1 * (−0.0498047128230464) + CASP3 * (−0.116381287112657) + CASP6 * (−0.283976042054973) + NLRP1 * 0.392333000687955 + GZMB * (−0.0829855594961543) + IRF1 * (−0.192571488474279). Furthermore, the exploration cohort was categorized into low- and high-risk groups based on the training dataset median value of the prognostic risk grade. The correlation between the six PRG signatures and the risk score can be observed in the heatmap, and the distribution of the risk score and survival time of CRC patients is displayed in the training and testing datasets (Supplementary Figures S3A, B). These results showed that the expression levels of CASP1, CASP3, CASP6, GZMB, and IRF1 were lower, while the expression levels of NLRP1 were higher in the high-risk groups than in the low-risk groups. In addition, the area under the curve (AUC) values for 1-, 3-, and 5-year OS were 0.70, 0.66, and 0.65 in the training dataset and were 0.67, 0.65, and 0.65 in the testing dataset, respectively (Supplementary Figures S3C, D). The results of the Kaplan–Meier (KM) curve analysis showed that the low-risk groups had a better prognosis than the high-risk groups in the training and testing datasets (*p* < 0.05) (Supplementary Figures S3E, F). Similarly, two external datasets including 177 patients in the GSE17536 cohort and 453 patients in the TCGA cohort were classified into the low- and high-risk groups based on the median risk score in the GSE39582 cohort (Figure 5A). The correlation between the six PRG signatures and the risk score can be observed in the heatmap (Figure 5B), and the distribution of the risk score and survival time of CRC patients were displayed in the two testing datasets (Figure 5C). In addition, the Kaplan-Meier analysis also indicated that patients in the low-risk groups have longer survival times than those in the high-risk groups (*p* < 0.05) (Figure 5D). ROC curve analysis of the two external datasets showed that our model had good predictive efficacy for 1- and 3-year OS; however, the AUC values for 5-year OS barely showed satisfaction (GSE17536: AUC = 0.64, 0.61, 0.58 for 1-, 3-, and 5-year survival; TCGA: AUC = 0.63, 0.62, and 0.56 for 1-, 3-, and 5-year survival, respectively) (Figure 5E). To further predict the ability of the prognostic model, the RFS was investigated to distinguish between low- and high-risk CRC patients. As predicted, the RFS differed between the low- and high-risk groups, implying that the PRGs signature had good accuracy in the prognostic prediction of CRC (*p* < 0.001) (Figure 5F). Subsequently, the PCA was performed to test the difference between the low- and high-risk groups based on the 38 PRGs and six PRG signatures. Notably, our results suggest that the low- and high-risk groups were better distinguished by the six PRG signatures than the 38 PRGs in the GSE39582 cohort, GSE17536 cohort, and TCGA cohort (Supplementary Figures S4A–H).

### Stratification Analysis and Independent Prognostic Value of the PRG Signature

**FIGURE 6:**
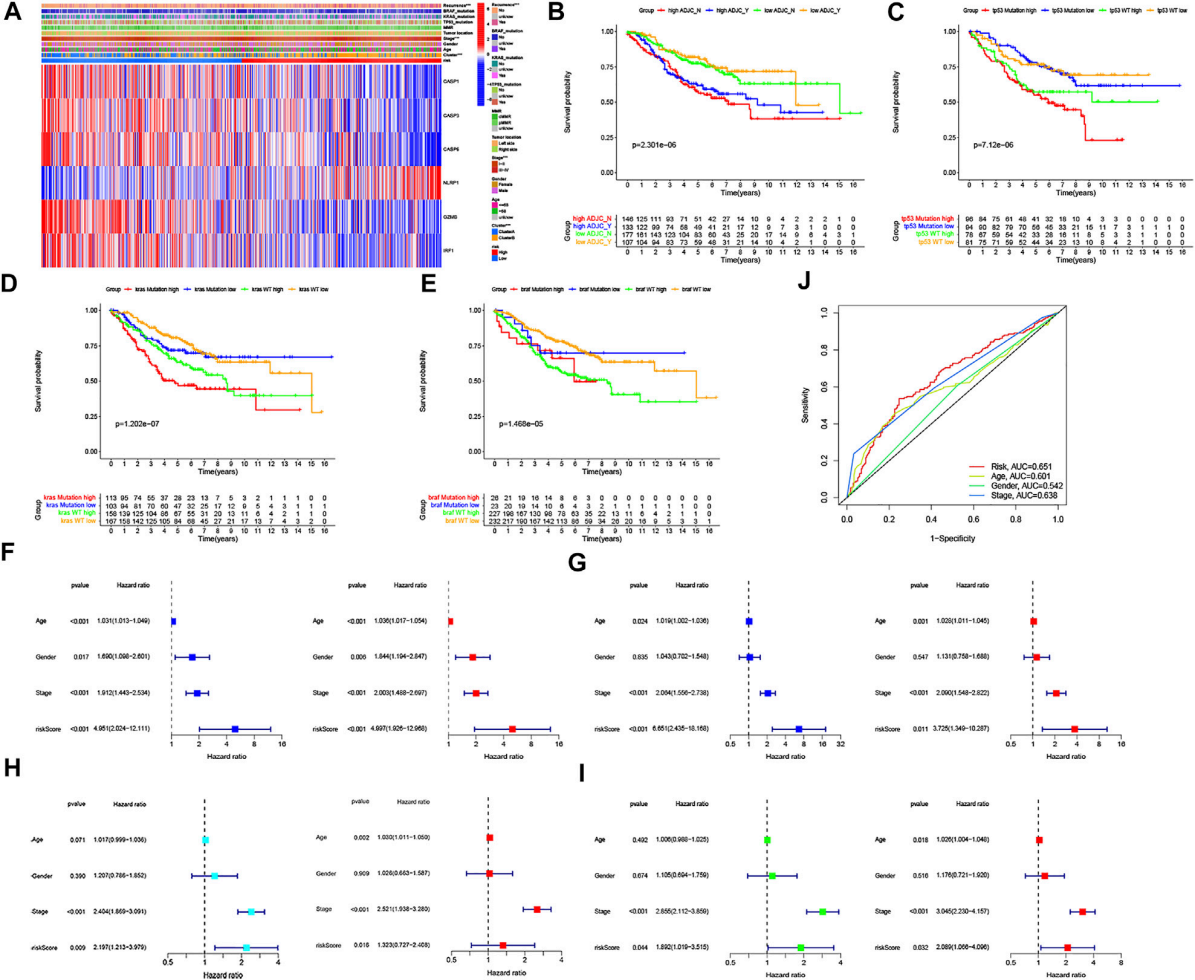
Association of six PRG signatures with clinical characteristics and the univariate and multivariate Cox regression analyses for the risk score. **(A)** Heatmap of the correlation of six PRG signatures with two CRC subtypes and clinical characteristics, ***p < 0.001. **(B–E)** Kaplan–Meier curve analysis of OS is shown for patients classified according to adjuvant chemotherapy and the TP53/KRAS/BRAF mutation status and six PRG signatures. **(F–I)** Univariate Cox regression and multivariate Cox regression analyses for the two internal (divided GSE39582 cohort into F: training dataset and G: testing dataset, respectively) and external datasets (H: TCGA cohort, **(I)** GSE17536 cohort, respectively). **(J)** ROC curves of the clinical characteristics and risk score.

To explore the impact of the PRG signature on clinical characteristics, we investigated the association between PRG signature and clinical factors in CRC patients, including age, gender, pathologic stage, TNM stage system, adjuvant chemotherapy, tumor location, MMR system, TP53 mutation status, KRAS mutation status, BRAF mutation status, and disease recurrence. As shown in Supplementary Figures S5A–M, we found that CRC patients in the high-risk groups tended to have a lower OS rate than that in the low-risk groups based on our different stratifications. These results suggested that the PRG signature could well predict the prognosis of CRC regardless of clinical features. In addition, The heatmap was also performed to demonstrate the significant differences in terms of the two pyroptosis-related subtypes, pathologic stage, and disease recurrence between the low- and high-risk groups (Figure 6A). Specifically, the ability of the PRG signature to predict the efficacy of adjuvant chemotherapy (defined as ADJC Yes/No) in CRC was assessed and found that the low-risk groups always showed a clear survival advantage regardless of whether chemotherapy. Moreover, the low-risk groups showed significant therapeutic advantages compared to high-risk groups, implying that the predictive ability of the PRG signature was not affected by ADJC (Figure 6B). As we know, TP53, KRAS, and BRAF mutation status are related to the sensitivity of chemotherapy and immunotherapy, as well as can be used as a prognostic marker in CRC (Carethers and Jung, 2015). Therefore, we further investigated whether the PRG signature could predict the OS outcome better than TP53, KRAS, and BRAF mutation status. Patients in the high-risk groups (respectively, defined as TP53 mutation/high and TP53 wt/high, KRAS mutation/high and KRAS wt/high, BRAF mutation/high and BRAF wt/high) had a worse clinical outcome than patients in the low-risk groups (respectively, defined as TP53 mutation/low and TP53 wt/low, KRAS mutation/low and KRAS wt/low, BRAF mutation/low and BRAF wt/low) (Figures 6C–E). Interestingly, patients in the high-risk groups with TP53 wt, KRAS wt, and BRAF wt had a worse OS outcome than patients in the low-risk groups with TP53 mutation, KRAS mutation, and BRAF mutation. However, patients in the high-risk groups with TP53 mutation, KRAS mutation, and BRAF mutation had a similar prognosis to that patient in the high-risk groups with TP53 wt, KRAS wt, and BRAF wt. Thus, these results suggested that the PRG signature might have greater prognostic significance than the TP53, KRAS, and BRAF mutation status. In addition, to determine whether the PRG signature could be used as independent prognostic factors in the two internal and external datasets, we attempted to perform univariate and multivariate Cox regression analyses based on the PRG signature and clinical features. As shown in Figures 6F–I, the PRG signature could act as independent predictors for the prognosis of CRC in GSE39582 cohort (two internal datasets), with consistent results observed in the GSE17536 cohort and TCGA cohort (two external datasets). The AUC values for 5-years OS were constructed by multivariate cox regression (*p* < 0.05). The AUC of the PRG signature was also higher than the other clinical characteristics (age, gender, and pathologic stage), suggesting that the PRG signature for the prediction prognosis of CRC was comparatively dependable (Figure 6J).

### Construction of a Nomogram for the Individualized Prediction Model in CRC

**FIGURE 7:**
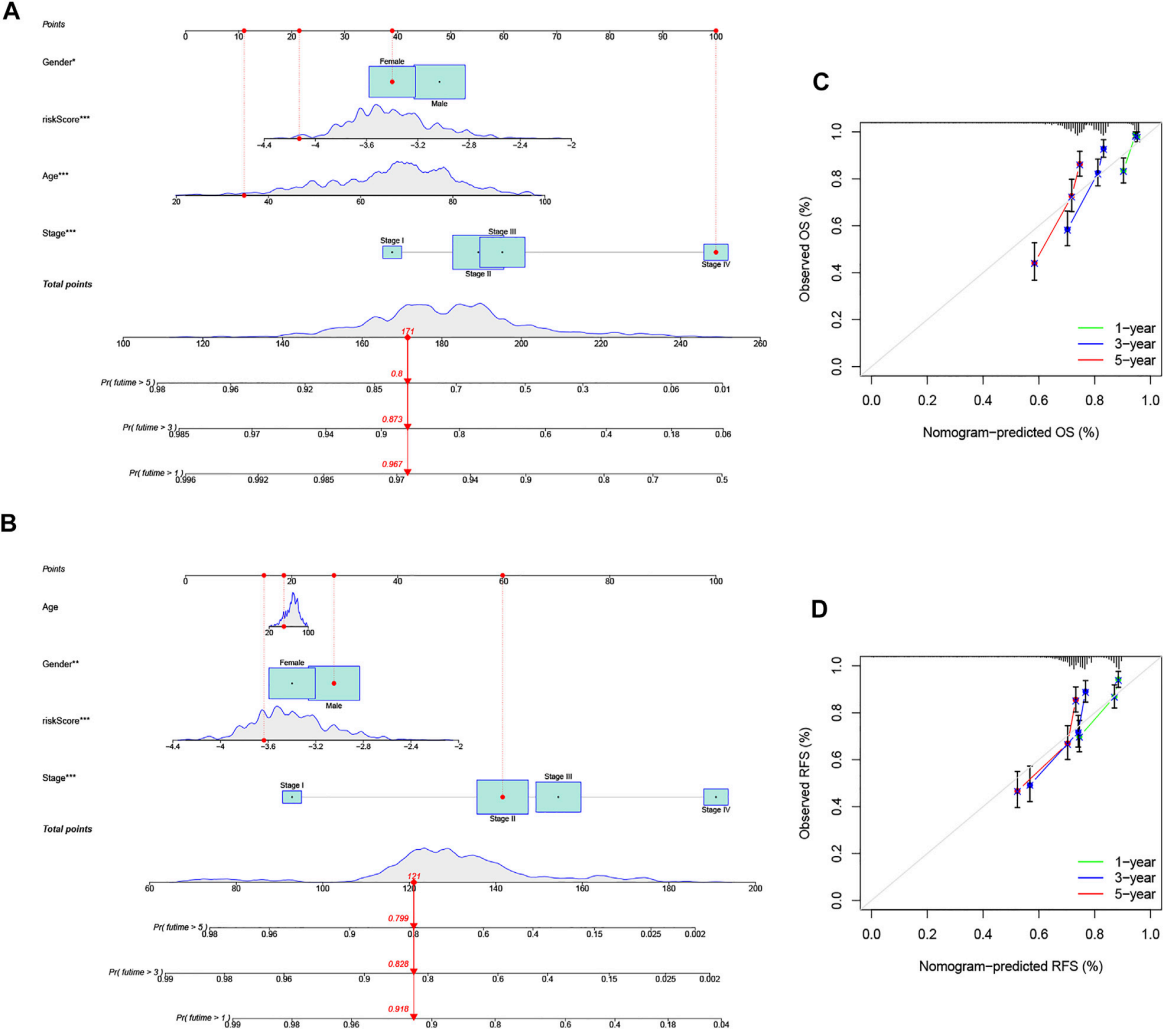
Construction and evaluation of a predictive nomogram. **(A)** Nomogram predicts the probability of OS at 1, 3, and 5 years. **(B)** Nomogram predicts the probability of RFS at 1, 3, and 5 years. **(C)** Calibration plot of the nomogram predicts the probability of the 1-, 3-, and 5-year OS. **(D)** Calibration plot of the nomogram predicts the probability of the 1-, 3-, and 5-year RFS.

Given the importance of the PRG signature in predicting the prognosis of CRC patients, we further attempted to construct a nomogram based on the multivariate Cox regression for predicting the OS and RFS at 1, 3, and 5 years. As shown in Figures 7A,B, the predominant predictive ability of the risk score in the nomogram was exhibited compared with the clinical characteristics, including age, gender, and pathologic stage. Moreover, the calibration plots indicated that the 1-, 3-, and 5-year OS and RFS could be predicted relatively well in the GSE39582 cohort (Figures 7C,D).

### Functional Enrichment Analysis of the PRG Signature in CRC

**FIGURE 8:**
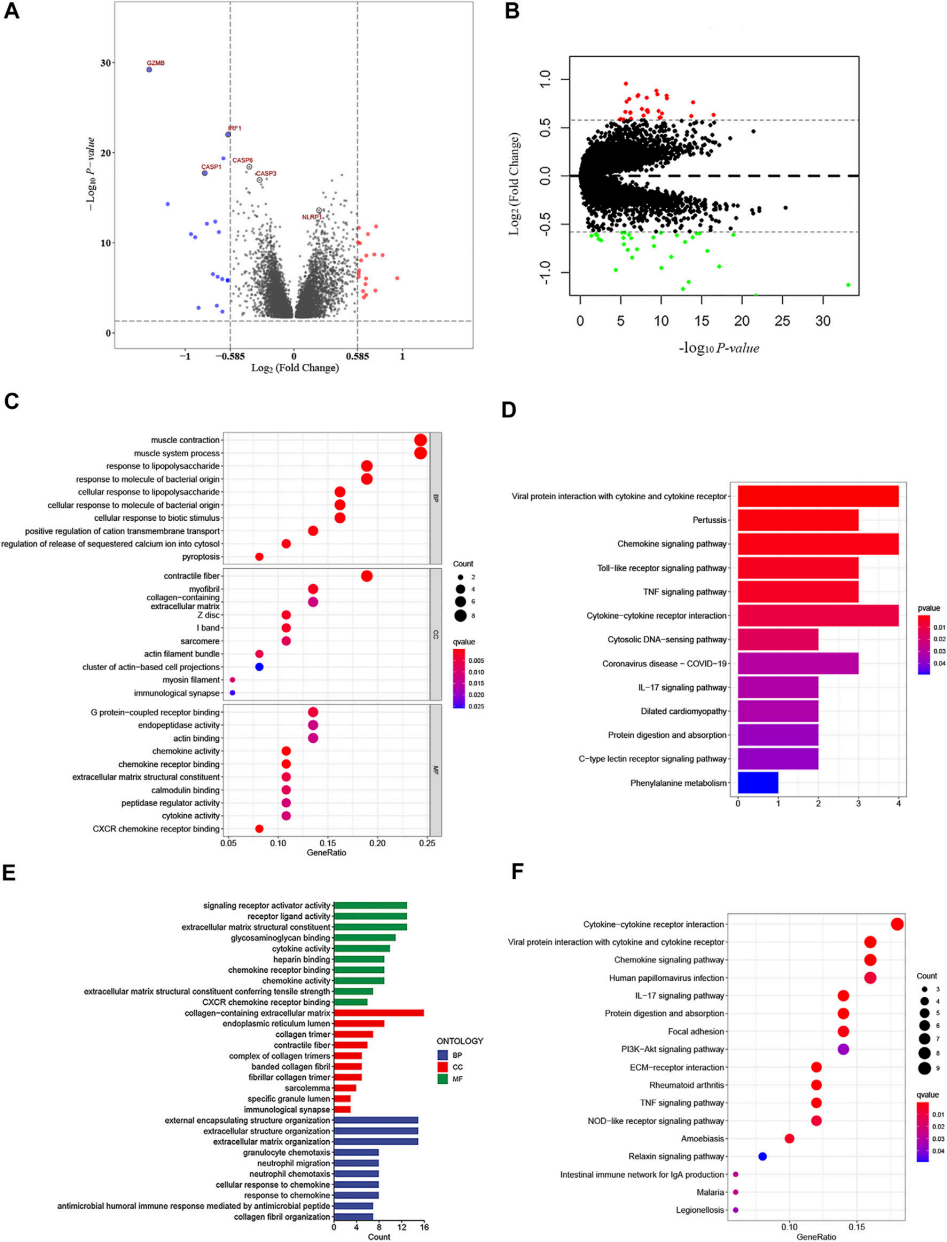
Functional analysis based on the DEGs between the high- and low-risk groups. **(A)** Overview of the DEGs between the two-risk groups in the GSE39582 cohort (red represents upregulated genes, blue indicates downregulated genes, and gray represents no change, respectively). **(B)** Volcano figure of significant DEGs between the two-risk groups in the TCGA cohort (Red represents upregulated genes, green indicates downregulated genes, and gray represents no change, respectively). **(C,D)** Bubble graph for gene ontology (GO) analysis and the barplot graph for Kyoto Encylopedia of Genes and Genomes (KEGG) analysis in the GSE39582 cohort. **(E,F)** Barplot graph for gene ontology (GO) analysis and the bubble graph for Kyoto Encylopedia of Genes and Genomes (KEGG) analysis in TCGA cohort.

To further explore the differences in the gene functions and pathways between the low- and high-risk groups based on PRG signature, we first identified 37 DEGs (FDR <0.05 and |Fold change| ≥ 1.5) between the low- and high-risk groups in the GSE39582 cohort. The volcano plot was applied to visually display the distribution of the DEGs and to depict the expression of the six significant variable PRG signature (Figure 8A, red: upregulated; blue: downregulated). Furthermore, we employed GO enrichment analysis and KEGG pathway analysis based on these DEGs. The results indicated that the PRG signature was mainly correlated with the immune response, chemokine-mediated signaling pathways, and inflammatory cell chemotaxis (Figures 8C,D). Similarly, 76 DEGs in the TCGA cohort were also identified between the low- and high-risk groups (Figure 8B, red: upregulated; green: downregulated). Interestingly, the results of functional enrichment analysis were consistent with the GSE39582 cohort (Figures 8E,F).

### Relationship Between the PRG Signature and TME Infiltration in CRC

**FIGURE 9:**
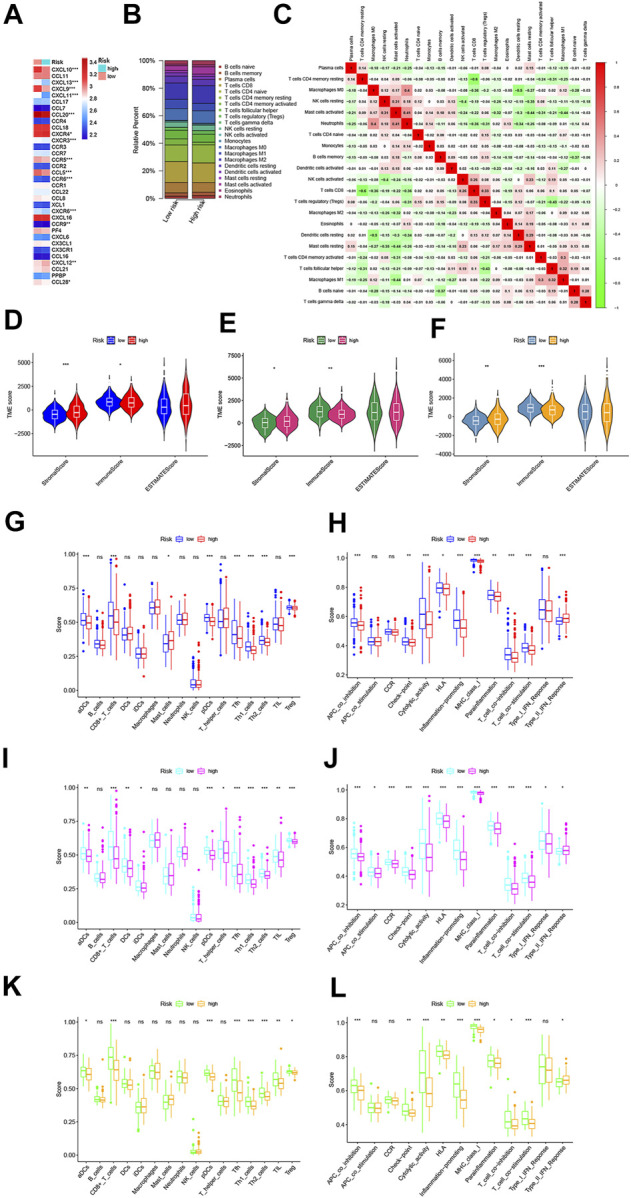
Variations in immune-related genes and the infiltration characteristics of TME cells between the high- and low-risk groups. **(A)** Thermogram shows variations in the mRNA expression of chemokines between the two-risk groups. **(B)** Bar plot shows the proportion of 22 infiltrating immune cells based on the CIBERSORT algorithm between the two-risk groups. **(C)** Correlation matrix of all 22 infiltrating immune cells (red represents positively related and green represents negatively related; *p* < 0.05 was the cut-off). **(D–F)** Correlations between the two-risk groups and TME score in GSE39582 **(D)**, TCGA **(E),** and GSE17536 **(F)** cohorts, respectively. **(G–L)** Comparison of the enrichment scores of 16 types of immune cells and 13 immune-related pathways between low- and high-risk groups in GSE39582 (G, H, respectively), TCGA [**(I, J)**, respectively] and GSE17536 [**(K, L)**, respectively] cohorts. *p < 0.05; **p < 0.01; and ***p < 0.001.

Increasing evidence reveals that TME cell infiltration is critical for carcinogenesis and the therapeutic response of tumors. Based on the functional analyses, we first analyzed the effect of chemokines on TME and found that there were higher expressions of CXCL9, CXCL10, CXCL12, CXCR4, and CCL5, in the low-risk group, which attract dendritic cells (DCs) and CD8 + T cells (Figure 9A). Furthermore, we investigated the abundance of different immune cell types between the low- and high-risk groups using CIBERSORT algorithm in GSE39582 cohort (Figure 9B). Heatmap showed the correlation between 22 immune cell types and numeric in each tiny box indicating the values of correlation between two kinds of immune cells in TME infiltration of the two risk groups (Figure 9C). The high-risk groups were closely associated with a high stromal score, whereas the low-risk groups were also significantly connected to a high immune score in both the GEO and TCGA cohorts (Figures 9D–F). In addition, we performed to explore the score of immune infiltration levels and immune functions between the low- and high-risk groups by using single-sample gene set enrichment analysis (ssGSEA). As shown in Figure 9G, the levels of immune cell infiltration in the high-risk groups were generally lower than those in the low-risk groups, especially for CD8+T cells, T helper (Th) cells (Tfh, Th1, and Th2 cells), and regulatory T (Treg) cells in the GSE39582 cohort. In addition, the immune-related functions, except for the type-II IFN response pathway, were also less active in the high-risk groups than in the low-risk groups (Figure 9H). A similar conclusion was reached when evaluating the immune status in the GSE17536 cohort and TCGA cohort. Moreover, we found that dendritic cells (DCs), induced dendritic cells (iDCs), T helper (Th) cells, tumor-infiltrating lymphocytes (TILs), co-stimulation of antigen-presenting cells (APCs), chemotaxis of CCR, and type-I INF response pathway were enriched in the low-risk groups compared with the high-risk groups (Figures 9I–L). Meanwhile, we discovered significant differences in immune cell infiltration between low- and high-risk groups in the GSE39582 cohort (Supplementary Figure S6A) such as the infiltration levels of CD4 memory-activated T cells, follicular helper T cells, M1 macrophages, and plasma cells were higher in the low-risk groups than those in the high-risk groups, while resting CD4 memory T cells, regulatory T cells (Tregs), monocytes, M2 macrophages, activated mast cells, and neutrophils had significantly lower infiltration in the low-risk groups than those in the high-risk groups. In the GSE17536 cohort, resting CD4 memory T cells, monocytes, and activated mast cells had higher infiltration levels in the high-risk groups, CD4 memory-activated T cells, follicular helper T cells, gamma delta T cells, and M1 macrophages had higher infiltration levels in the low-risk groups (Supplementary Figure S6B). In TCGA cohort, we found that the infiltration levels of CD8 + T cells, CD4 memory-activated T cells, activated NK cells, M1 macrophages, resting mast cells, and eosinophils were higher in the low-risk groups than those in the high-risk groups, while regulatory T cells (Tregs), M0 and M2 macrophages, and activated mast cells had significantly lower infiltration in the low-risk groups compared to those in the high-risk groups (Supplementary Figure S6C). Moreover, we generated a scatter diagram to visually display the association between our risk model and the abundance of immune cells. As shown in Supplementary Figures S7A–D, the risk score was positively correlated with memory B cells, Tregs, resting CD4 memory T cells, monocytes, M0 and M2 macrophages, neutrophils, activated mast cells and negatively correlated with CD8 + T cells, CD4 memory-activated T cells, gamma delta T cells, follicular helper T cells, plasma cells, M1 macrophages, resting mast cells, and activated NK cells. Taken together, our results suggested that the PRG signature plays an important role in the immune regulation of the TME.

### Relationship of the PRG Signature With Mutation, MSI, CSC Index, and EMT

**FIGURE 10:**
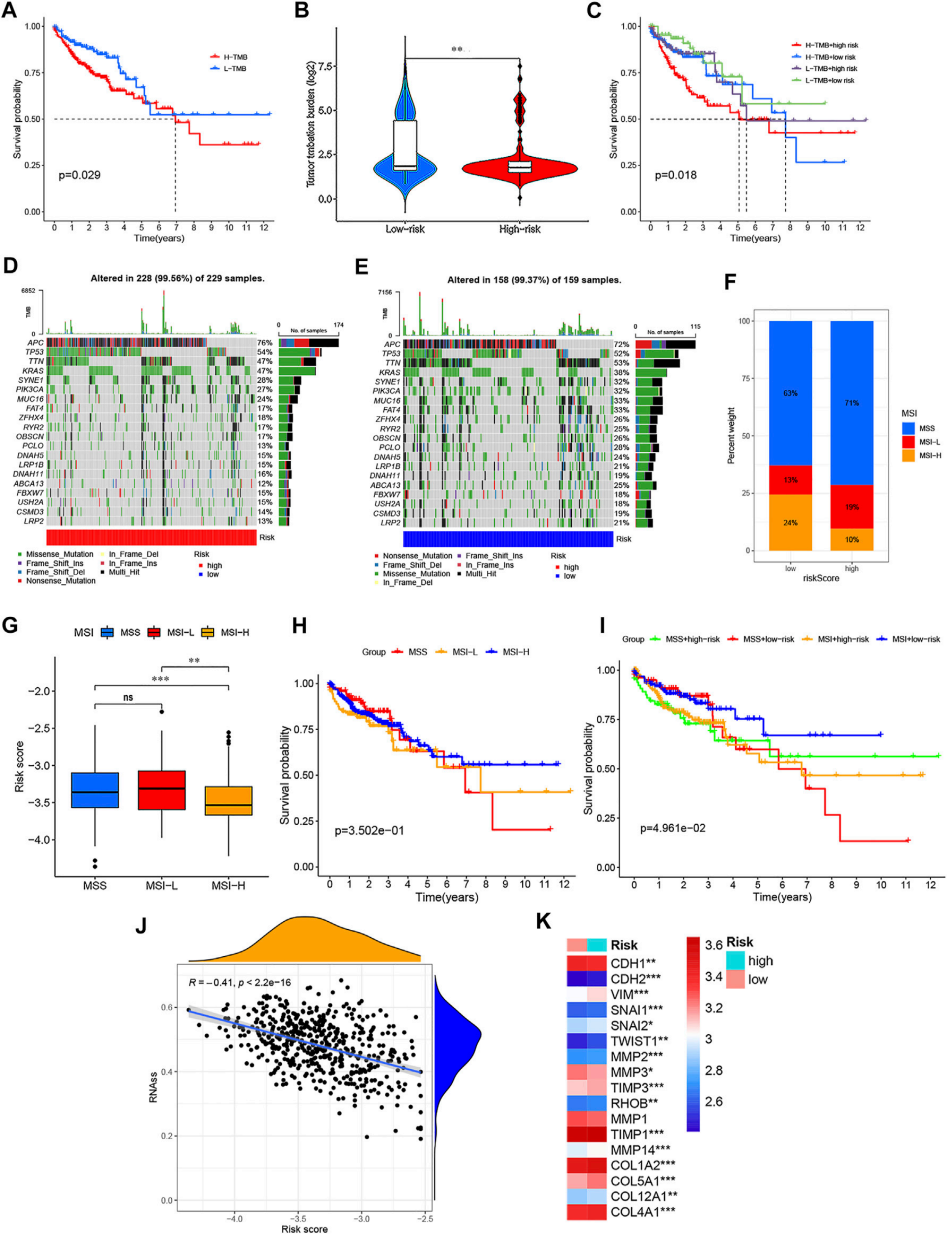
Comprehensive analysis of the PRG signature in CRC. **(A)** Kaplan–Meier curves for the high- and low-TMB with the cut-off value of 2.421 of 399 CRC patients in TCGA cohorts. **(B)** TMB difference in the high- and low-risk groups. **(C)** Kaplan–Meier curves for CRC patients in TMB and risk score subgroups. **(D,E)** Waterfall plot of somatic mutation features established with the high- and low-risk groups. **(F,G)** Correlation between two risk groups and MSI. **(H)** Kaplan–Meier curves for MSS, MSI-L, and MSI-H of CRC patients. **(I)** Kaplan–Meier curves for CRC patients in MSS, MSI-L, and MSI-H and risk score subgroups. **(J)** Correlation between two risk groups and CSC index. **(K)** Thermogram shows variations in the mRNA expression of EMT-related genes between the two risk groups. *p < 0.05; **p < 0.01; and ***p < 0.001.

**FIGURE 11:**
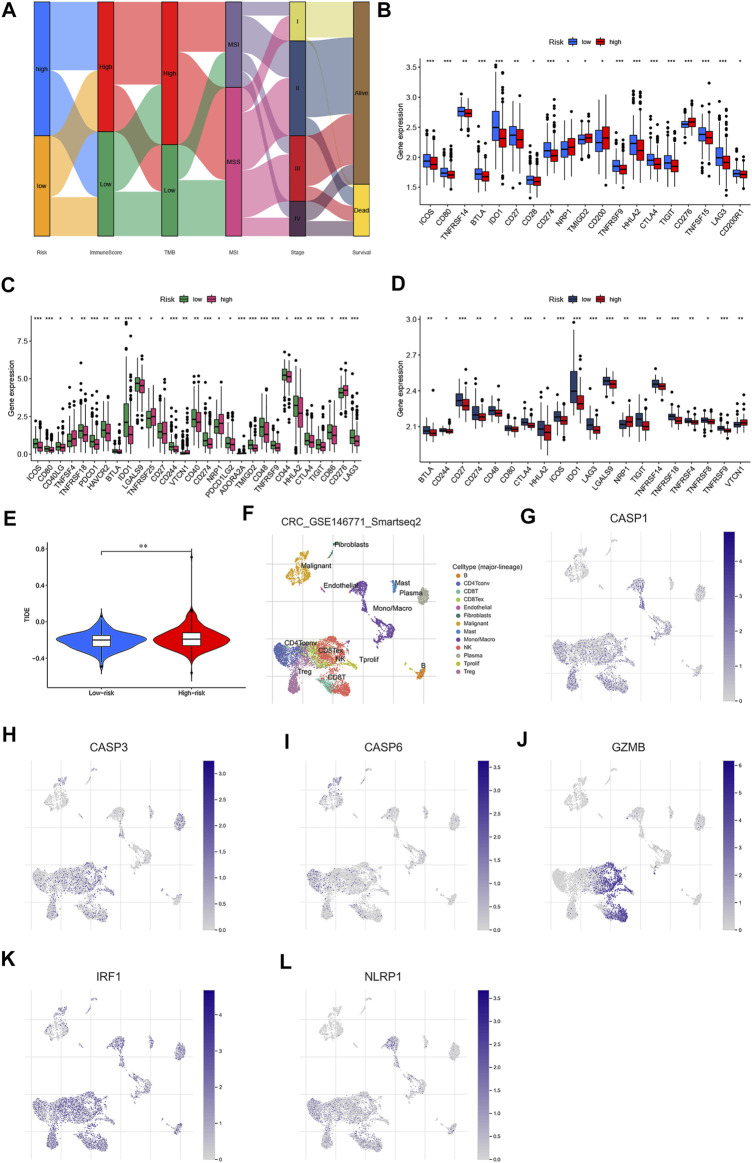
Estimation and validation of the 47 immune checkpoints and cancer immunotherapy response in two risk groups. **(A)** Alluvial diagram showing the changes in risk score, immune score, TMB, MSI, TMN staging, and survival outcomes in the GSE39582 cohort. Expression of immune checkpoints between the high- and low-risk groups in GSE39582 **(B)**, TCGA **(C)**,and GSE17536 **(D)** cohorts, respectively. **€** TIDE prediction difference in the high- and low-risk groups. **(F–L)** Correlation and distribution of the expressed PRG signature and immune cell infiltration in CRC. *p < 0.05; **p < 0.01; and ***p < 0.001.

Based on the TCGA-COAD/READ mutation data, we first assessed the potential relationship between the TMB level and OS and observed that the survival rate of patients with high TMB was lower than that of patients with low TMB (the optimal cutoff = 2.4211; *p* = 0.029) (Figure 10A). In addition, Figure 10B showed a lower TMB in the low-risk groups than that in high-risk groups, and the survival rate of the low-risk groups was more affected by TMB status than that of the high-risk groups (*p* = 0.018) (Figure 10C). Furthermore, the distribution variations of the top ten somatic mutation genes between two PRG signature groups were analyzed based on the TCGA-COAD cohort (Figures 10D,E). Except for the APC, TP53, and KRAS, other mutated genes had higher mutation frequencies in the low-risk groups than the high-risk groups. Moreover, correlation analyses revealed that the high-risk groups were associated with microsatellite stability (MSS) and low microsatellite instability (MSI−L) status, while low-risk groups were correlated with the high microsatellite instability (MSI-H) status (Figures 10F,G). Although the influence of MSI status on OS in CRC is less obvious, we found that patients with MSS status had a poorer prognosis than the other two subgroups (*p* = 0.3502) (Figure 10H). Interestingly, combination of the PRG signature risk model and MSI/MSS (respectively defined as MSI + high-risk, MSI + low-risk, MSS + high-risk, and MSS + low-risk) could clearly stratify patients better. As shown in Figure 10I, patients with MSS + low-risk group had worse survival outcomes than patients with MSI + low-risk group, while the trend of survival advantage in the MSI + high-risk group was reversed by the risk score (*p* < 0.05). Moreover, we analyzed the relationships between the risk score and cancer stem cell (CSC) in the two risk groups. As shown in Figure 10J, the result demonstrated a negative correlation between the risk score and the CSC index values (R = −0.41; *p* < 0.001), implying that the low-risk score had more distinct stem cell properties and a lower degree of cell differentiation. EMT is one of the core mechanisms of tumor metastasis, and it is also one of the main factors leading to poor prognosis of patients. Notably, we found that EMT markers, including N-cadherin, vimentin, snails, TWIST1, and MMPs, were higher in high-risk groups than in low-risk groups (Figure 10K). Finally, a sankey diagram was constructed to visualize these changes in patient characteristics (Figure 11A).

### PRG Signature Predict Sensitivity of CRC to Immunotherapy and Chemotherapy

There is increasing evidence suggesting that patients with high TMB and MSI-H status may benefit from immune checkpoint blockade therapy (Topalian et al., 2016; Ganesh et al., 2019; Picard et al., 2020; Zhang et al., 2020). We then performed to analyze the associations between 47 important immune checkpoints (Mahoney et al., 2015; Wang J. B et al., 2020; Zhang et al., 2020; Zhao et al., 2021) and our risk model in the GSE39582 cohort. As shown in Figures 11B–D, we found that immune checkpoints (showed statistical differences among 47 immune checkpoints) were differentially expressed between low- and high-risk groups in GSE39582, GSE17536, and TCGA datasets, including PD-L1, CTLA-4, and LAG3. According to these results, we speculated that low-risk groups might benefit from immunotherapy compared to high-risk groups. Unsurprisingly, we found that the high-risk groups showed higher dysfunction and exclusion score by using the Tumor Immune Dysfunction and Exclusion (TIDE) algorithm, implying that the low-risk groups benefited from immunotherapy (Figure 11E), consistent with the results observed in the MSS analyses. To explore the potential therapeutic drugs of patients in the high-risk groups, we further performed the analysis of the drug sensitivity (IC50 value) based on Genomics of Drug Sensitivity in Cancer (GDSC). According to the drug result, we found that the IC50 of most drugs, such as cisplatin, gemcitabine, and camptothecin were significantly lower in low-risk groups than those in high-risk groups, while IC50 of pazopanib, midostaurin, imatinib, elesclomol, dasatinib, bryostatin.1, bicalutamide, bexarotene, etc., were significantly lower in the patients in high-risk groups (Supplementary Figure S8), suggesting that our study might provide therapeutic schedules for further analysis. Together, our PRG signature model is a potential metric for evaluating prognoses and the clinical response to immunotherapy and chemotherapy.

### Correlation of the PRG Signature Expression With Immune Infiltration and Survival in CRC

**FIGURE 12:**
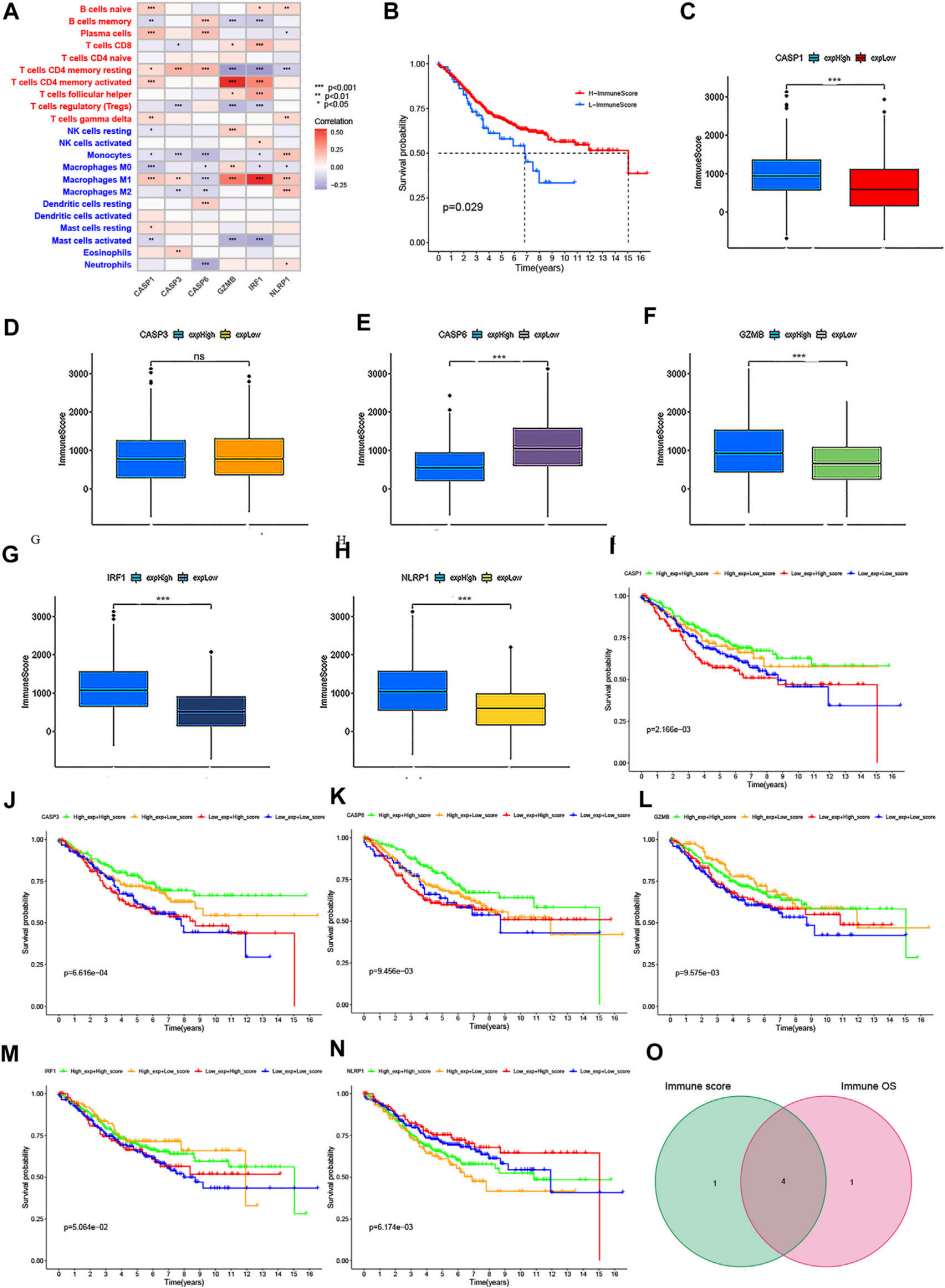
Correlation of the immune score with the OS of patients in CRC. **(A)** Relationships between the abundance of 22 types of immune cells and six PRG signatures. **(B)** Kaplan–Meier curves for the high- and low-immune score with the cut-off value -11.072 of 579 CRC patients. **(C–H)** Relationships between the expression levels of six PRG signatures and immune score with the cut-off value -11.072 of 579 CRC patients. **(I–N)** Kaplan–Meier curves for CRC patients in immune score and PRG signature expression subgroups. **(O)** Venn plot showed four common PRG signatures based on both immune score and prognosis. *p* < 0.05 was the cut-off. *p < 0.05; **p < 0.01; and ***p < 0.001.

To identify the roles of PRG signature in the TME of CRC, we used the TISCH database to analyze the distribution of the PRG signature in CRC. We found that the expression distributions of CASP1, CASP3, GZMB, and NLRP1 were abundant in immune cells. The gene expression of CASP6 and IRF1 were evenly distributed in immune cells and malignant cells (Figures 11F–L). We also analyzed the distribution of PRG signature in immune infiltration. The results showed that most immune cells were significantly associated with the PRG signature (Supplementary Figure S9), which was consistent with previous results. (Figure 12A). We further analyzed the relationship between PRG signature expression and staging in different subsets of cells. Except for the CD8 + T cells, six PRGs signatures were significantly correlated with staging in the mononuclear/macrophage and plasma cells subgroup (Supplementary Figure S10). To explore the correlation of the estimated proportion of immune infiltration with the OS rate, we performed Kaplan–Meier survival analysis based on the level of ImmuneScore. As shown in Figure 12B, the proportion of immune components had a positive correlation with the OS rate (*p* = 0.029). Furthermore, we investigated the differences in terms of Immune scores with high-/low-expression groups depending on the comparison with the median expression levels of six-PRG signatures. Except for the CASP3, other PRG signatures had a significant correlation with the ImmuneScore (*p* < 0.001) (Figures 12C–H). The effect of gene expression combined with the level of immune infiltration on the prognosis of CRC had been focused on. As shown in Figures 12I–N, the OS was longer in high CASP1/CASP3/CASP6/GZMB expression (respectively, defined as High_exp and Low_exp) + high ImmuneScore (respectively, defined as High_score and Low_score) and low NLRP1 expression + high ImmuneScore, while the IRF1 expression combined with the level of ImmuneScore had no significant correlation with the OS. Finally, four common PRG signatures (CASP1, CASP6, NLRP1, and GZMB) were identified from the two lists (Figure 12O), which were of ImmuneScore (*p* < 0.05) and immune-related OS (*p* < 0.05). We further conducted to verify the relationship between the expression of four PRG signatures and the levels of TME infiltration based on TIMER 2.0. The expression levels of CASP1 were found to be positively correlated with the infiltration levels of CD8 + T cells, plasma cells, dendritic cells (DCs), CD4 + T cells, monocytes, mast cells, NK cells, macrophages, regulatory T cells (Tregs), and neutrophils. Notably, we also found that the CASP1 expression was negatively associated with the cancer-associated fibroblast cells and tumor purity (Supplementary Figure S11A). In addition, there was a negative association between CASP6 expression and the immune infiltration level of CD8 + T cells, NK cells, macrophages, monocytes, dendritic cells (DCs), neutrophils, and cancer-associated fibroblast cells, whereas showed a positive association with the abundance of CD4 + T cells, plasma cells, and tumor purity (Supplementary Figure S11B). Moreover, the expression levels of GZMB were found to be positively correlated with the infiltration levels of CD8 + T cells, plasma cells, dendritic cells (DCs), CD4 + T cells, monocytes, NK cells, macrophages, neutrophils, endothelial cells, and cancer-associated fibroblast cells were negatively associated with regulatory T cells (Tregs) (Supplementary Figure S11C). Moreover, highly expressed NLRP1 was positively correlated with the infiltration level of plasma cells, CD4 + T cells, CD8 + T cells, macrophages, dendritic cells (DCs), NK cells, monocytes, neutrophils, regulatory T cells (Tregs), mast cells, endothelial cells, and cancer-associated fibroblast cells, while was negatively associated with tumor purity (Supplementary Figure S11D). These results indicated that PRGs had crosstalk between CRC and immune cells and had the potential to shape the unique TME of CRC.

### CASP6 and NLRP1 Had Potential to be Targets of Immunotherapy and Chemotherapy in CRC

**FIGURE 13:**
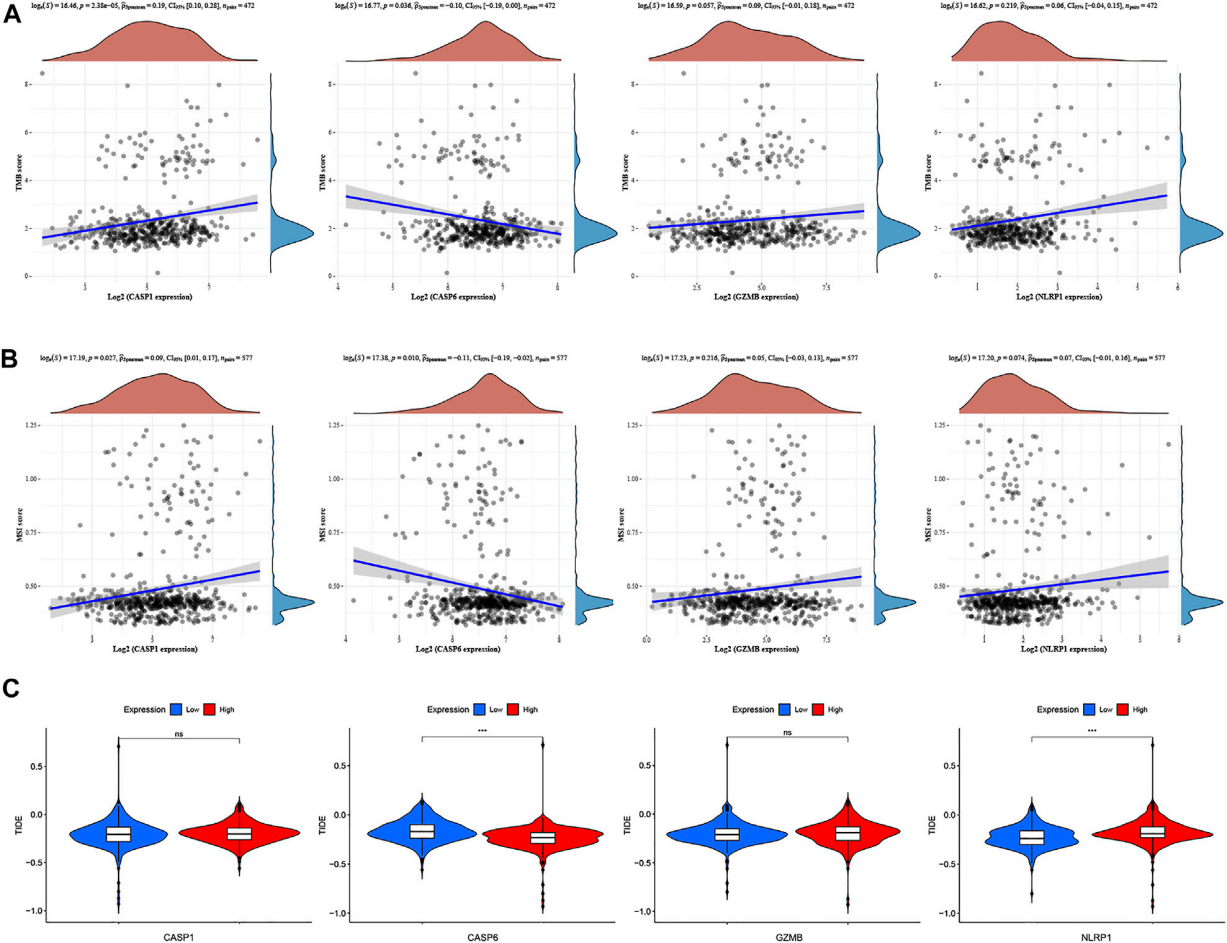
TMB, MSI, and cancer immunotherapy response analysis with four PRG signatures. **(A)** Correlation between four PRG signatures and TMB in CRC. **(B)** Relationships between four PRG signatures and MSI. **(C)** Relationship between four PRG signatures and TIDE.

The aforementioned results revealed that the expression levels of CASP1/CASP6/GZMB/NLRP1 significantly affected the immune activity of TME in CRC. To clarify whether these PRGs could also serve as biomarkers to predict the response to immune checkpoint blockade therapy, we first detected the correlation of several important immune checkpoints (such as PD-L1, LAG3, and CTLA-4) with them in GSE39582 cohort. As shown in Supplementary Figures S12A–C, the data demonstrated a negative correlation between the CASP6 expression and the immune checkpoints, while other PRGs were positively correlated with the immune checkpoints (*p* < 0.05). Furthermore, Spearman's correlation analysis demonstrated that the expression levels of CASP1 were positively associated with a high TMB (*p* = 2.38e−05), while CASP6 expression was negatively related to a high TMB (*p* = 0.036) (Figure 13A). In addition, the results revealed a positive correlation between CASP1 and MSI (*p* = 0.027), whereas MSI was negatively correlated with CASP6 expression (*p* = 0.01) (Figure 13B). There was no significant correlation between GZMB and TMB or MSI (*p* = 0.057 and *p* = 0.216, respectively). Interestingly, we found that low CASP6 expression showed higher dysfunction and a lower exclusion score using the TIDE algorithm (*p* < 0.001), suggesting that high CASP6 expression may benefit from immunotherapy in CRC (Figure 13C). This is inconsistent with the results of TMB and MSI, which might be related to the negative correlation between CASP6 expression and the immune checkpoints. Despite NLRP1 expression had no significant correlation with the TMB or MSI (*p* = 0.219 and *p* = 0.074, respectively), our results indicated that low NLRP1 expression also might benefit from immunotherapy in CRC (*p* < 0.001). To validation of the expression levels of CASP6 and NLRP1 used for therapeutic targets, RT-qPCR was carried out in six pairs of CRC tissues and normal tissues. As shown in Supplementary Figures S12D–E, compared with normal tissues, the expressions of CASP6 and NLRP1 in CRC tissues were significantly lower. The experimental results were consistent with the results predicted by bioinformatics methods (Supplementary Figure S2) and GEPIA database (Supplementary Figures S12F, G). In Human Protein Atlas (HPA) database, the expression levels of CASP6 were downregulated in CRC tissues compared to the levels in the corresponding normal tissues (Supplementary Figures S12H, I). However, there was no significant difference in the expression of NLRP1 (Supplementary Figures S12J, K). Also, the verification in the GEPIA database found that the low expression of CASP6 and high expression of NLRP1 had a higher survival risk, and the expression of CASP6 and NLRP1 were significantly associated with the stages of CRC, which were consistent with our results (Supplementary Figure S13). In addition, CRC recurrence is attributed to chemoresistance. Thus, we further focused on the correlation of CASP6 and NLRP1 expressions with the sensitivities of chemotherapy drugs that currently were used for the treatment of CRC. Interestingly, the IC50 values of shikonin, cisplatin, paclitaxel, gefitinib, and camptothecin showed a positive association with the expression levels of CASP6, while the correlation of IC50 values of 5-Fu and gemcitabine with CASP6 expression was negative. Moreover, we also found that the IC50 values for shikonin, cisplatin, paclitaxel, and camptothecin were significantly negatively associated with the expression levels of NLRP1, and the IC50 values of gemcitabine showed a positive association with the expression levels of NLRP1. However, the IC50 values of 5-Fu and gefitinib had no significant correlation with the NLRP1 expression (Supplementary Figure S14). 5-Fu chemoresistance is a major challenge and the prognosis for CRC patients can be very poor due to recurrence of the disease (Brenner et al., 2014). For further validation, the pcDNA3.1-CASP6 expression plasmid was transfected into HT29 and HCT116 cells. CASP6 expression was drastically increased in both HCT116 and HT29 cells after transfection with the pcDNA3.1-CASP6 vector compared with the expression in cells transfected with an empty pcDNA3.1 vector (Supplementary Figures S15A, B). Elevation in CASP6 significantly inhibited the proliferation ability of HT29 and HCT116 cells (Supplementary Figures S15C, D). In addition, our experiments’ results suggested that pcDNA3.1-CASP6 increased cytotoxicity induced by 5-Fu in HCT116 and HT29 cells (Supplementary Figure S15E, F). Moreover, the IC50 value of 5-Fu in HCT116 and HT29 cells were approximately 40 and 30 μM, and over-expression of CASP6 also increased IC50 value of 5-FU induced apoptosis in HCT116 cells and HT29 cells compared with controls (Supplementary Figure S15G). Therefore, these results indicated that low expression of CASP6 and high expression of NLRP1 promoted tumor development, and it also was associated with poor prognosis, the sensitivity of immunotherapy, and chemotherapy in CRC.

### CASP6 Induces Chemoresistance of CRC Cells to 5-Fu Partly Associated With *Fusobacterium nucleatum*

**FIGURE 14:**
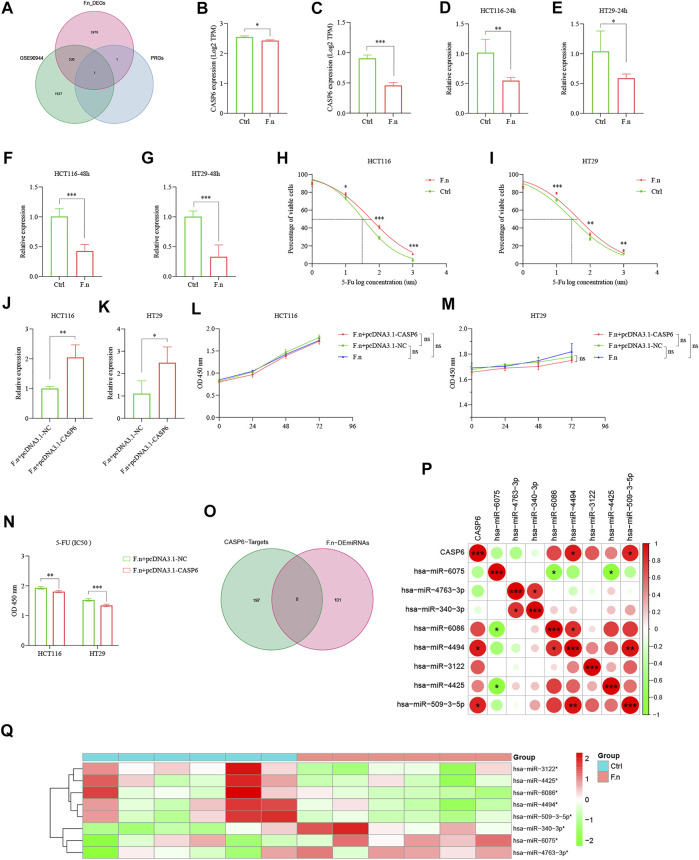
*F.n* induces chemoresistance of CRC cells to 5-Fu *via* downregulation of CASP6 *in vitro*. **(A)** Venn diagram showed CASP6 shared by *F.n*-related DEGs and PRG signature. **(B)** Expression of CASP6 in GSE90944. **(C)** Expression of CASP6 between HCT116 cells and *F.n*-infected HCT116 cells using microarray analysis. **(D–G)** Expression of CASP6 was quantified by qPCR in CRC cell lines infected with or without *F.n* for 24 and 48 h, respectively. **(H,I)** Cells were incubated with or without *F.n* and then exposed to serial dilutions of 5-Fu for 48 h (cell viability was determined by CCK8 assay, and data are presented as the percentage of viable cells). **(J,K)** RT–qPCR analysis of the CASP6 expression in CRC cell lines co-cultured with *F.n* for 24 h and then transfected with pcDNA3.1-CASP6 or empty pcDNA3.1 vector. **(L, M)** Cell proliferation was detected in HCT116 cells and HT29 cells after transfection with pcDNA3.1-CASP6 or empty pcDNA3.1 vector. **(N)** Cell viability of HCT116 cells and HT29 cells were detected using a CCK8 assay after transfection with a CASP6 overexpression vector under *F.n* treatment for 24 h. **(O)** Venn diagram of differential miRNAs and possibly combined miRNAs with CASP6. **(P, Q)** Heatmap of significantly differential eight candidates' miRNAs **(Q)** and relationships between CASP6 and eight candidates' miRNAs (P). *p < 0.05; **p < 0.01; and ***p < 0.001.

The important contribution of the gut microbiota to human health and disease is widely recognized. Increasing evidence suggests that patients with high *F.n* abundance are associated with poor RFS and can promote chemoresistance to 5-Fu in CRC (33, 34). We hypothesized that dysregulated *F.n* might contribute to downregulate the CASP6 expression. To test this hypothesis, we performed a global mRNA expression profiling of HCT116 cell lines infected with *F.n* for 24 h (Supplementary Table S5). Additionally, we analyzed the gene expression profile of the RNA-seq dataset (GSE90944) in HT29 cell lines treated with or without *F.n*. Interestingly, we found there could be a possible association between *F.n* and CASP6 (Figure 14A, DEGs was set as an FDR <0.05 and |log_2_ FC| ≥ 1). In addition, the results showed that the mRNA levels of CASP6 were significantly downregulated by *F.n* (Figure 14B: GSE90944, Figure 14C: mRNA Chip array). Furthermore, we performed to validate the downregulation of CASP6 in both HCT116 and HT29 cells infected with *F.n* for 24 and 48 h based on qPCR (Figures 14D–G). To determine whether the downregulation of CASP6 expression induced by *F.n* infection leaded to chemoresistance in CRC, we first detected the cytotoxicity induced by 5-Fu (Absin, China) in CRC cells with or without *F.n* infection. The results suggested that *F.n* decreased cytotoxicity induced by 5-Fu in HCT116 and HT29 cells (Figures 14H,I). Moreover, pcDNA3.1-CASP6 was transfected in HCT116 cells and HT29 cells cultured with *F.n* (Figures 14J,K), and we found that had no effect on CRC proliferation (Figures 14L, M). However, pcDNA3.1-CASP6 increased IC50 5-FU-induced apoptosis in HCT116 cells and HT29 cells cultured with *F.n*, compared with controls (Figure 14N). miRNAs often regulate gene expression by binding to the RNA-induced silencing complex (RISC) and directing sequence-specific cleavage of target mRNA or repressing the target mRNA translation (Krek et al., 2005; Bartel, 2009). We speculated that dysregulated miRNAs might contribute to downregulate CASP6 expression by *F.n*. To test this hypothesis, 252 miRNAs were identified that can bind to the 3′-UTR of CASP6 based on miRWalk database analysis (Supplementary Table S6). In addition, a total of 109 differentially expressed miRNAs (*p* < 0.05) were identified by using miRNA data, which is presented in the Supplementary Material (miRNA expression profiling of twelve CRC tissues with a distinct amount of *F.n*). Finally, eight common miRNAs were identified from these two lists, and we speculated that the hsa-miR-4494 and hsa-miR-509-3-5p might regulate *F.n*-CASP6-chemoresistance axis (Figures 14O–Q, Supplementary Table S7). Further study should be conducted to verify these results. Taken together, our results proved that gut microbiota dysregulation could affect the expression of CASP6 and thus induce chemoresistance of CRC cells to 5-Fu.

## Discussion

There is increasing evidence suggesting the indispensable role of pyroptosis in innate immunity and antitumor effects (Zaki et al., 2010; Wang Q et al., 2020; Tsuchiya, 2021). However, there is a lack of bioinformatics analysis to demonstrate the immune infiltration characteristics and prognostic values of pyroptosis in CRC. In this study, all the pathways directly related to pyroptosis were explored and a prognostic signature was identified by analyzing the influence of the involved pathways on the TME infiltration. Our PRG signature provided potential targets for immunotherapy of pyroptosis and implied that pyroptosis combined with immunotherapy to improve the prognosis of patients might be an effective treatment direction. Moreover, this study is the first to suggest that pyroptosis may be involved in the interactions between gut microbiota and the chemo-sensitivity to 5-Fu in CRC. We hope that our findings will help improve our understanding of the role of pyroptosis and provide a foundation for future research targeting pyroptosis to improve treatment design and the accuracy of prognosis in CRC.

First, 585 cases of CRC from GSE39582 datasets were selected as the exploration cohort, and two distinct molecular subtypes were identified based on the expression levels of 38 PRGs. The characteristics of clinical and TME infiltration differed significantly between the two subtypes. Two subtypes were also characterized by a significant immune activation, suggesting that there was a significant correlation between pyroptosis levels and the TME infiltration in CRC. Second, the PGR signature model was constructed to quantify the prognostic risk in both GEO and TCGA cohorts by using LASSO penalized Cox regression analysis. Patients with low- and high-risk groups showed significantly different prognoses, clinicopathological characteristics, TME infiltration, TMB, MSI, CSC index, immune checkpoints, TIDE, and drug susceptibility. Notably, there was a negative correlation between the high-risk groups and the infiltration level of anti-tumor immune cells. Moreover, we confirmed that the PGR signature could be used for prognosis stratification of patients with CRC, and the predictive ability of the risk model was reliable. Moreover, multivariate Cox regression analysis indicated that the PRG signature could act as an independent predictor. Finally, we established a quantitative nomogram to improve the performance and facilitated the use of the model by integrating the PRG signature and clinical characteristics. Taken together, our PGR signature will assist in better understanding the molecular mechanism of CRC and may provide dependable molecular biomarkers for CRC therapies.

A large number of studies have revealed the importance of the immune microenvironment in tumorigenesis. Our results implied that the immune components in the TME contributed to the prognosis of CRC patients. Here, we revealed that the expression levels of CASP1, CASP6, GZMB, and NLRP1 were significantly associated with the proportion of immune components in the TME and prognosis (expression combined with ImmuneScore). In addition, a low CASP6 expression and high NLRP1 expression showed higher dysfunction and a lower exclusion score based on the TIDE algorithm, which implied that CASP6 and NLRP1 might be a potential prognostic marker and a therapeutic target for the immune microenvironment in CRC. Previous studies have confirmed that PRGs play a crucial role in chemotherapy (Jiang et al., 2020; An et al., 2021; Shen et al., 2021). 5-Fu chemoresistance is a major challenge and the prognosis for CRC patients can be very poor due to recurrence of disease (Brenner et al., 2014). Thus, we focused on CASP6 and NLRP1 to determine whether it was related to 5-Fu sensitivity. Interestingly, the expression levels of CASP6 had a negative correlation with the IC50 value for 5-Fu (*p* < 0.001). CASP6 has been revealed that is involved in carcinogenesis and progression by facilitating the activation of programmed cell death pathways, including pyroptosis, apoptosis, and necroptosis (Lee et al., 2006; Cheng et al., 2016; Chen et al., 2021; Lin et al., 2021). Recently, CASP6 has been generally considered to be a key regulatory factor for innate immune-inflammatory activation and host defense (Zheng et al., 2020). Combining the importance of CASP6 effects on the immune microenvironment, our results suggested the possibility of using target CASP6 to enhance the immunotherapy and chemotherapy effect on CRC. In addition, *F.n*, an anaerobic bacterium parasitic in the oral cavity, is increasingly linked to colorectal carcinogenesis and therapies (Rubinstein et al., 2013; Yang et al., 2017; Yu et al., 2017; Zhang S et al., 2019). However, there are no reports on the influence of the gut microbiota on the progression of CRC mediated by the expression of pyroptosis. Astonishingly, we investigated the results of sequencing obtained after co-culture of *F.n* with colon cancer cell lines and determined that the expression of CASP6 was significantly decreased in both HCT116 and HT29 cell lines. Thus, *F.n* might promote 5-Fu resistance by reducing the expression of CASP6, and affect the prognosis of CRC patients. Furthermore, we performed to combine these data with the data on differential expression of miRNAs in CRC tissues with a distinct amount of *F.n*, which indicated that changes in the expression levels of miRNAs acting on the corresponding 3′-UTR of CASP6. According to this result, *F.n* might influence the changes in CASP6 at the transcriptional and posttranscriptional levels and further influence the progression and the prognosis of CRC. Further study verification of the mechanism may identify new pathogenic pathways and therapeutic targets.

Nonetheless, our study had several limitations. Most of the results were predicted by bioinformatics analysis, and the transcriptional level could reflect the TME infiltration status but not reflect the specific changes. We should pay attention to the results of some single-cell sequencing in the future, which may be able to explain the specific changes in the TME. In addition, multicenter clinical queues should be performed to verify our results. Our results suggested that CASP6 might play an important role in CRC; therefore, further experiments *in vitro* or *in vivo* were needed to demonstrate the associations between pyroptosis, gut microbiota, and CRC. Our laboratory is conducting further research on the subject.

## Conclusion

In conclusion, we systematically analyzed the expression and prognostic value of pyroptosis in CRC and provided a thorough evaluation of the heterogeneity and complexity of the TME. Low expressions of certain PRGs could be used as a molecular marker to identify CRC patients in high-risk groups. The therapeutic liabilities of pyroptosis in immunotherapy and chemotherapy were also determined. At present, this is the first research to propose the theory that gut microbiota may influence chemoresistance of CRC cells in response to 5-Fu by influencing the transcriptional changes in pyroptosis, which provides a direction for further study. These findings highlight the vital clinical implications of pyroptosis and provide new ideas for guiding personalized therapeutic strategies for CRC patients.

## Data Availability

The original contributions presented in the study are included in the article/Supplementary Material; further inquiries can be directed to the corresponding author.
