# Supplementary material for: Pyroptosis-Related Signature as Potential Biomarkers for Predicting Prognosis and Therapy Response in Colorectal Cancer Patients
Source: Front Genet. 2022 Jul 22;13:925338. doi: 10.3389/fgene.2022.925338 (PMC9355164; doi:10.3389/fgene.2022.925338)
Supplement: Supplementary file 3 [file Table1.DOCX]

Supplementary Table 1. 38 pyroptosis-related genes

| Genes | Full-names |
| --- | --- |
| AIM2 | Absent in melanoma 2 |
| CASP1 | cysteine-aspartic acid protease-1 |
| CASP3 | cysteine-aspartic acid protease-3 |
| CASP4 | cysteine-aspartic acid protease-4 |
| CASP5 | cysteine-aspartic acid protease-5 |
| CASP6 | cysteine-aspartic acid protease-6 |
| CASP8 | cysteine-aspartic acid protease-8 |
| CASP9 | cysteine-aspartic acid protease-9 |
| ELANE | elastase, neutrophil expressed |
| GPX4 | glutathione peroxidase 4 |
| GSDMA | gasdermin A |
| GSDMB | gasdermin B |
| GSDMC | gasdermin C |
| GSDMD | gasdermin D |
| GSDME | gasdermin E |
| IL18 | interleukin 18 |
| IL1B | interleukin 1 beta |
| IL6 | interleukin 6 |
| NLRC4 | NLR family CARD domain containing 4 |
| NLRP1 | NLR family pyrin domain containing 1 |
| NLRP2 | NLR family pyrin domain containing 2 |
| NLRP3 | NLR family pyrin domain containing 3 |
| NLRP6 | NLR family pyrin domain containing 6 |
| NLRP7 | NLR family pyrin domain containing 7 |
| NOD1 | nucleotide binding oligomerization domain containing 1 |
| NOD2 | nucleotide binding oligomerization domain containing 2 |
| PJVK | pejvakin/deafness, autosomal recessive 59 |
| PLCG1 | phospholipase C gamma 1 |
| PRKACA | protein kinase cAMP-activated catalytic subunit alpha |
| PYCARD | PYD and CARD domain containing |
| SCAF11 | SR-related CTD associated factor 11 |
| TIRAP | TIR domain containing adaptor protein |
| TNF | tumor necrosis factor |
| GZMA | granzyme A |
| GZMB | granzyme B |
| HMGB1 | high mobility group box 1 |
| IRF1 | interferon regulatory factor 1 |
| IRF2 | interferon regulatory factor 2 |

Supplementary Table 2. Primers of genes and sequences of mRNA mimics

| **Primer sequence** | **Forward** | **Reverse** |
| --- | --- | --- |
| CASP6 | 5’-CAACGCAGACAGAGACAACCT-3’ | 5’-TCGACACCTCGTGAATTTTGAG-3’ |
| β-actin | 5’-TCACCCACACTGTGCCCATCTACGA-3’ | 5’-CAGCGGAACCGCTCATTGCCAATGG-3’ |
| **Sequence of mimics** | **Sense (5'-3')** | **Antisense (5'-3')** |
| CASP6 mimics | CTAGAGAACCCACTGCTTAC | TAGAAGGCACAGTCGAGG |
| Control mimics | UUCUCCGAACGUGUCACGUTT | ACGUGACACGUUCGGAGAATT |

Supplementary Table 3 univariate Cox regression analysis of 37 PRGs associated with OS in CRC patients

| Genes | HR | HR.95L | HR.95H | P-value | Km |
| --- | --- | --- | --- | --- | --- |
| AIM2 | 0.934844 | 0.808564 | 1.080846 | 0.362841 | 0.035671 |
| CASP1 | 0.84181 | 0.741242 | 0.956022 | 0.007983 | 0.000476 |
| CASP3 | 0.727451 | 0.56392 | 0.938405 | 0.014313 | 0.000648 |
| CASP4 | 1.282274 | 0.988724 | 1.662977 | 0.060866 | 0.007502 |
| CASP5 | 0.879092 | 0.765266 | 1.009848 | 0.068538 | 0.013322 |
| CASP6 | 0.677197 | 0.534668 | 0.857722 | 0.001226 | 0.000267 |
| CASP8 | 0.832992 | 0.578223 | 1.200013 | 0.326567 | 0.091693 |
| CASP9 | 0.832025 | 0.485543 | 1.425756 | 0.503373 | 0.087505 |
| ELANE | 1.252714 | 0.533762 | 2.940056 | 0.604713 | 0.165781 |
| GPX4 | 1.0438 | 0.817423 | 1.33287 | 0.731085 | 0.063312 |
| GSDMB | 0.862766 | 0.698047 | 1.066355 | 0.172063 | 0.00852 |
| GSDMC | 1.795926 | 0.948438 | 3.400696 | 0.072264 | 0.030102 |
| GSDMD | 0.816094 | 0.631827 | 1.054101 | 0.119604 | 0.030385 |
| GSDME | 1.222758 | 0.998371 | 1.497577 | 0.051871 | 0.012627 |
| IL18 | 0.89529 | 0.767097 | 1.044905 | 0.160664 | 0.028871 |
| IL1B | 1.03477 | 0.925076 | 1.157471 | 0.549969 | 0.075831 |
| NLRC4 | 1.640884 | 0.926773 | 2.905241 | 0.089307 | 0.007815 |
| NLRP1 | 1.517004 | 1.083201 | 2.124539 | 0.015307 | 0.000413 |
| NLRP2 | 1.039421 | 0.891683 | 1.211638 | 0.621095 | 0.067856 |
| NLRP3 | 1.407117 | 0.916188 | 2.161106 | 0.118731 | 0.0383 |
| NLRP6 | 0.799976 | 0.61114 | 1.047161 | 0.104265 | 0.065306 |
| NLRP7 | 0.906658 | 0.4698 | 1.749741 | 0.770195 | 0.140942 |
| NOD2 | 0.990658 | 0.800312 | 1.226275 | 0.931291 | 0.038692 |
| PLCG1 | 1.178877 | 0.898576 | 1.546615 | 0.234853 | 0.070806 |
| PYCARD | 0.920051 | 0.774238 | 1.093327 | 0.343905 | 0.035695 |
| GZMA | 0.927752 | 0.822843 | 1.046035 | 0.220629 | 0.010709 |
| GZMB | 0.869855 | 0.78722 | 0.961163 | 0.006186 | 0.000149 |
| HMGB1 | 1.004548 | 0.808977 | 1.247398 | 0.967239 | 0.119542 |
| IRF1 | 0.796791 | 0.651799 | 0.974035 | 0.026645 | 0.001245 |
| IRF2 | 0.951259 | 0.69555 | 1.300978 | 0.754424 | 0.058158 |
| IL6 | 1.092266 | 0.970214 | 1.229673 | 0.144347 | 0.013308 |
| NOD1 | 1.329242 | 0.882093 | 2.003059 | 0.173728 | 0.013277 |
| PJVK | 1.306728 | 0.887033 | 1.924999 | 0.175899 | 0.004724 |
| PRKACA | 1.281449 | 0.801061 | 2.049921 | 0.300867 | 0.029174 |
| SCAF11 | 1.081874 | 0.807471 | 1.449527 | 0.598032 | 0.146205 |
| TIRAP | 1.052702 | 0.623202 | 1.778205 | 0.847729 | 0.133782 |
| TNF | 1.119966 | 0.768171 | 1.632869 | 0.555892 | 0.14007 |

Supplementary Table 4 univariate Cox regression analysis of 37 PRGs associated with RFS in CRC patients

| Genes | HR | HR.95L | HR.95H | P-value | Km |
| --- | --- | --- | --- | --- | --- |
| AIM2 | 0.855474 | 0.729447 | 1.003274 | 0.054888 | 0.005353 |
| CASP1 | 0.818184 | 0.71877 | 0.931348 | 0.002397 | 0.001902 |
| CASP3 | 0.674338 | 0.524026 | 0.867766 | 0.002197 | 0.000209 |
| CASP4 | 1.133287 | 0.866209 | 1.482712 | 0.361507 | 0.02805 |
| CASP5 | 0.867562 | 0.749949 | 1.00362 | 0.05596 | 0.016819 |
| CASP6 | 0.670473 | 0.524454 | 0.857149 | 0.001423 | 0.001258 |
| CASP8 | 0.683331 | 0.471724 | 0.98986 | 0.044024 | 0.008621 |
| CASP9 | 0.932386 | 0.532058 | 1.633926 | 0.806772 | 0.012407 |
| ELANE | 1.059156 | 0.438516 | 2.558201 | 0.898356 | 0.073954 |
| GPX4 | 0.999128 | 0.776669 | 1.285306 | 0.994586 | 0.065369 |
| GSDMB | 0.671313 | 0.536308 | 0.840302 | 0.000504 | 0.000537 |
| GSDMC | 1.044235 | 0.539851 | 2.019865 | 0.897683 | 0.14087 |
| GSDMD | 0.766793 | 0.587241 | 1.001243 | 0.051076 | 0.006348 |
| GSDME | 1.31244 | 1.064947 | 1.617451 | 0.010767 | 0.000844 |
| IL18 | 0.856594 | 0.730809 | 1.00403 | 0.05609 | 0.003315 |
| IL1B | 0.929369 | 0.823711 | 1.048579 | 0.234204 | 0.081468 |
| NLRC4 | 0.774104 | 0.406157 | 1.475383 | 0.436512 | 0.000794 |
| NLRP1 | 1.249447 | 0.86577 | 1.803154 | 0.234099 | 0.005389 |
| NLRP2 | 1.100266 | 0.941647 | 1.285603 | 0.228979 | 0.000887 |
| NLRP3 | 1.050083 | 0.653421 | 1.687541 | 0.839994 | 0.054456 |
| NLRP6 | 0.797485 | 0.603371 | 1.054051 | 0.111818 | 0.060433 |
| NLRP7 | 0.529482 | 0.260941 | 1.074386 | 0.078199 | 3.26E-05 |
| NOD2 | 1.109994 | 0.89152 | 1.382007 | 0.350738 | 0.069757 |
| PLCG1 | 1.24246 | 0.942859 | 1.637262 | 0.123066 | 0.004612 |
| PYCARD | 1.04192 | 0.865152 | 1.254805 | 0.665071 | 0.323901 |
| GZMA | 0.834675 | 0.735016 | 0.947846 | 0.005343 | 0.002422 |
| GZMB | 0.876689 | 0.790439 | 0.97235 | 0.012752 | 0.000156 |
| HMGB1 | 1.05719 | 0.839485 | 1.331353 | 0.636407 | 0.195703 |
| IRF1 | 0.623435 | 0.503796 | 0.771486 | 1.38E-05 | 2.30E-06 |
| IRF2 | 0.87482 | 0.642996 | 1.190225 | 0.394563 | 0.01704 |
| IL6 | 0.980088 | 0.860869 | 1.115818 | 0.76118 | 0.088619 |
| NOD1 | 1.143223 | 0.748152 | 1.746917 | 0.53609 | 0.105164 |
| PJVK | 1.434123 | 0.970576 | 2.119061 | 0.07029 | 0.006823 |
| PRKACA | 1.001352 | 0.615435 | 1.629262 | 0.99566 | 0.144442 |
| SCAF11 | 1.070727 | 0.785293 | 1.45991 | 0.665731 | 0.040195 |
| TIRAP | 0.863484 | 0.500357 | 1.490146 | 0.598036 | 0.031951 |
| TNF | 0.928292 | 0.623176 | 1.382798 | 0.714399 | 0.034097 |
