# Supplementary material for: Pyroptosis-Related Signature as Potential Biomarkers for Predicting Prognosis and Therapy Response in Colorectal Cancer Patients
Source: Front Genet. 2022 Jul 22;13:925338. doi: 10.3389/fgene.2022.925338 (PMC9355164; doi:10.3389/fgene.2022.925338)
Supplement: Supplementary file 4 [file DataSheet1.PDF]

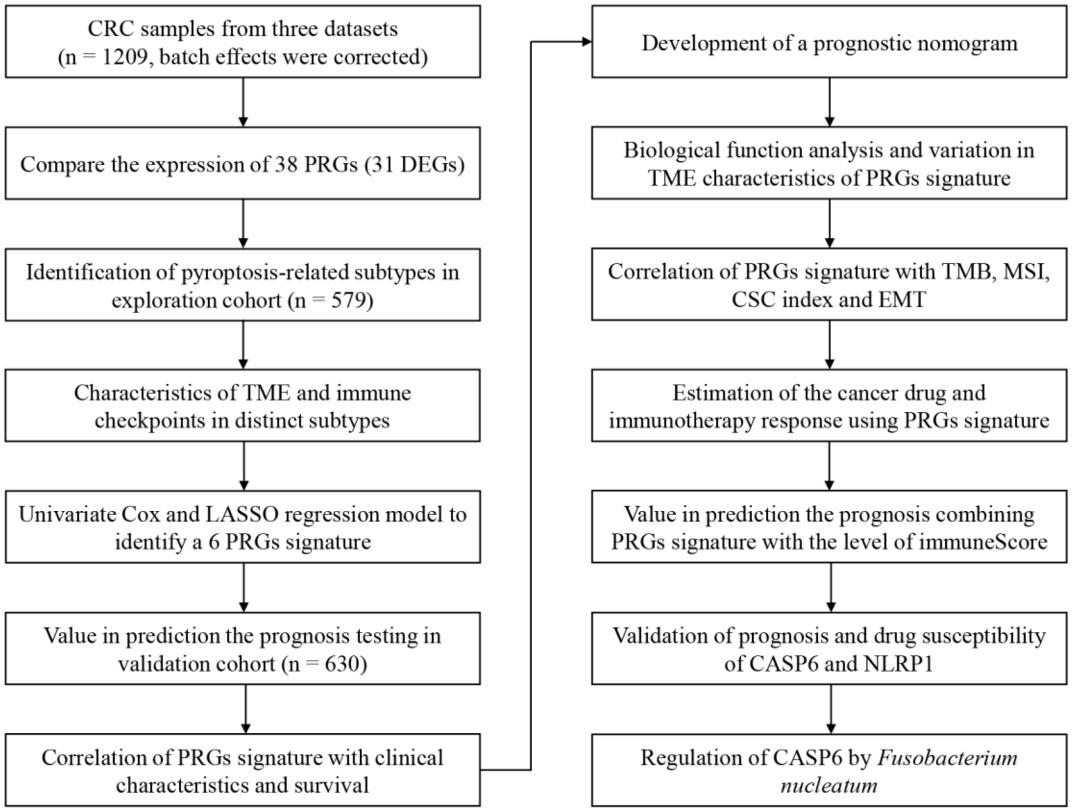
CRC samples from three datasets (n = 1209, batch effects were corrected)
Compare the expression of 38 PRGs (31 DEGs)
Identification of pyroptosis-related subtypes in exploration cohort (n = 579)
Characteristics of TME and immune checkpoints in distinct subtypes
Univariate Cox and LASSO regression model to identify a 6 PRGs signature
Value in prediction the prognosis testing in validation cohort (n = 630)
Correlation of PRGs signature with clinical characteristics and survival
Development of a prognostic nomogram
Biological function analysis and variation in TME characteristics of PRGs signature
Correlation of PRGs signature with TMB, MSI, CSC index and EMT
Estimation of the cancer drug and immunotherapy response using PRGs signature
Value in prediction the prognosis combining PRGs signature with the level of immuneScore
Validation of prognosis and drug susceptibility of CASP6 and NLRP1
Regulation of CASP6 by *Fusobacterium nucleatum*

Figure S1

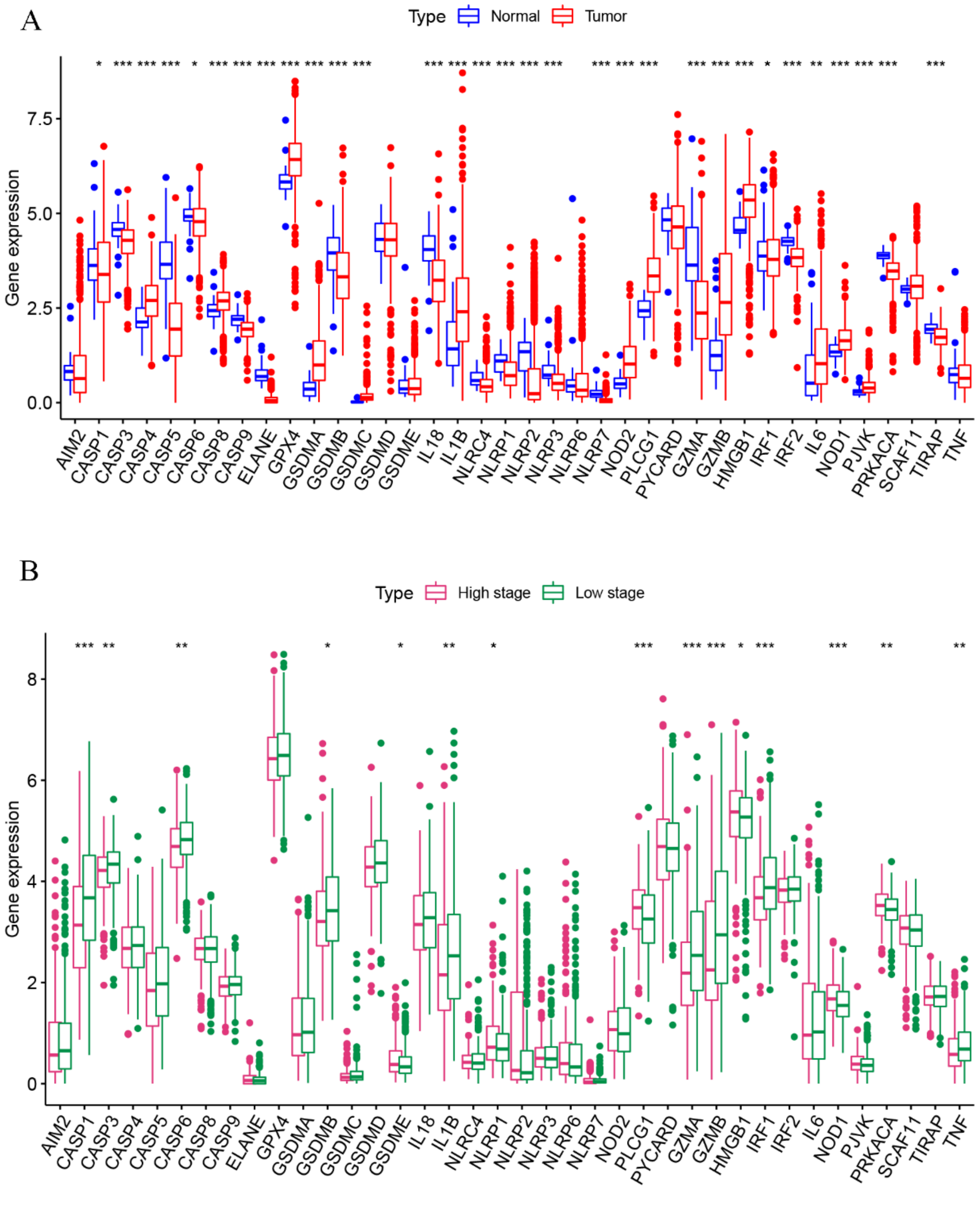

A
Type Normal Tumor
Gene expression
B
Type High stage Low stage
Gene expression
AIM2
CASP1
CASP3
CASP4
CASP5
CASP6
CASP8
CASP9
ELANE
GPX4
GSDMA
GSDMB
GSDMC
GSDMD
GSDME
IL18
IL1B
NLRC4
NLRP1
NLRP2
NLRP3
NLRP6
NLRP7
NOD2
PLCG1
PYCARD
GZMA
GZMB
HMGB1
IRF1
IRF2
IL6
NOD1
PJVK
PRKACA
SCAF11
TIRAP
TNF

Figure S2

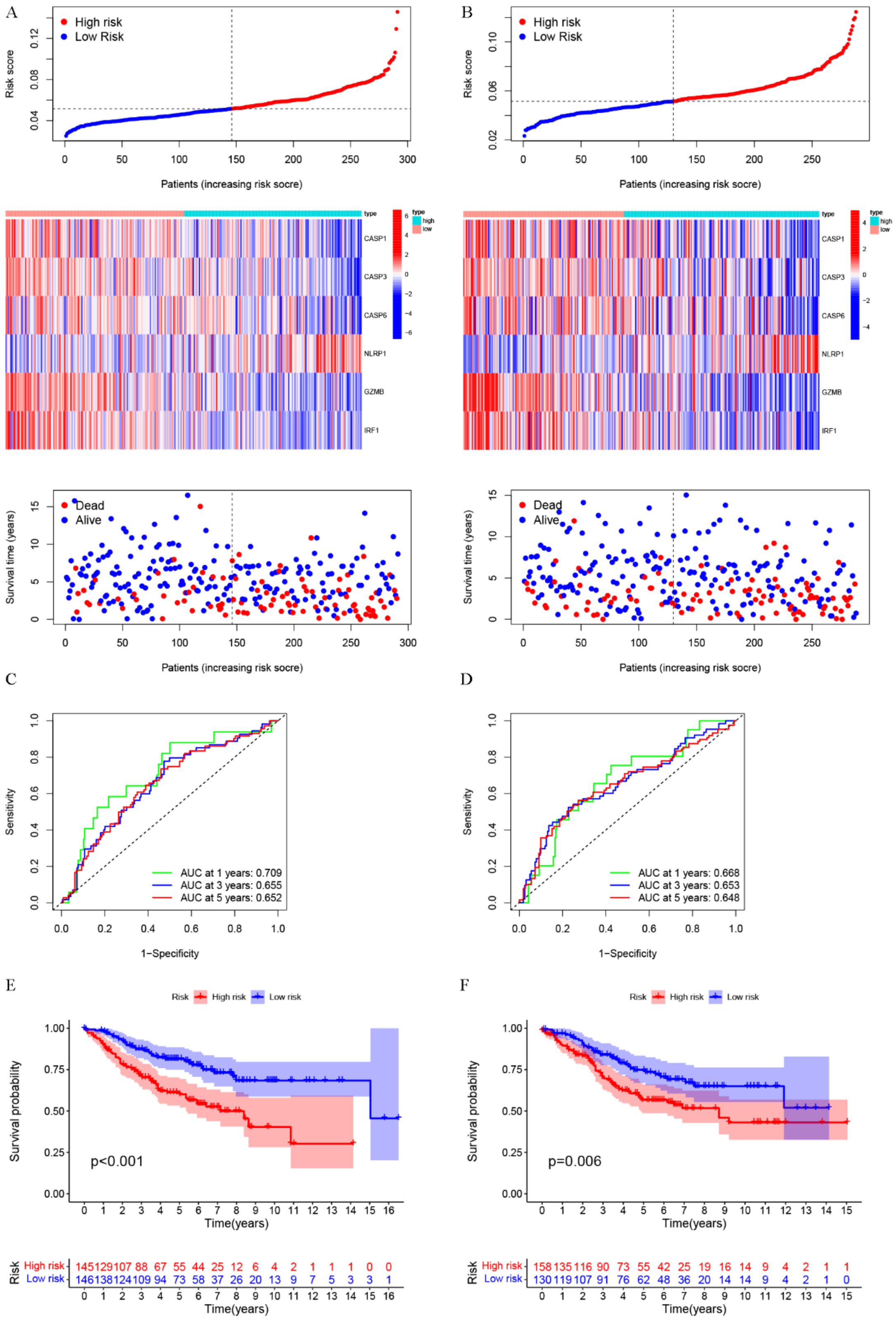
A
B
High risk
Low Risk
Risk score
Patients (increasing risk socre)
type
high
low
CASP1
CASP3
CASP6
NLRP1
GZMB
IRF1
Dead
Alive
Survival time (years)
C
D
Sensitivity
1−Specificity
AUC at 1 years: 0.709
AUC at 3 years: 0.655
AUC at 5 years: 0.652
AUC at 1 years: 0.668
AUC at 3 years: 0.653
AUC at 5 years: 0.648
E
F
Risk
High risk
Low risk
Survival probability
Time(years)
p<0.001
p=0.006
High risk 145 129 107 88 67 55 44 25 12 6 4 2 1 1 1 0 0
Low risk 146 138 124 109 94 73 58 37 26 20 13 9 7 5 3 3 1
High risk 158 135 116 90 73 55 42 25 19 16 14 9 4 2 1 1
Low risk 130 119 107 91 76 62 48 36 20 14 14 9 4 2 1 0

Figure S3

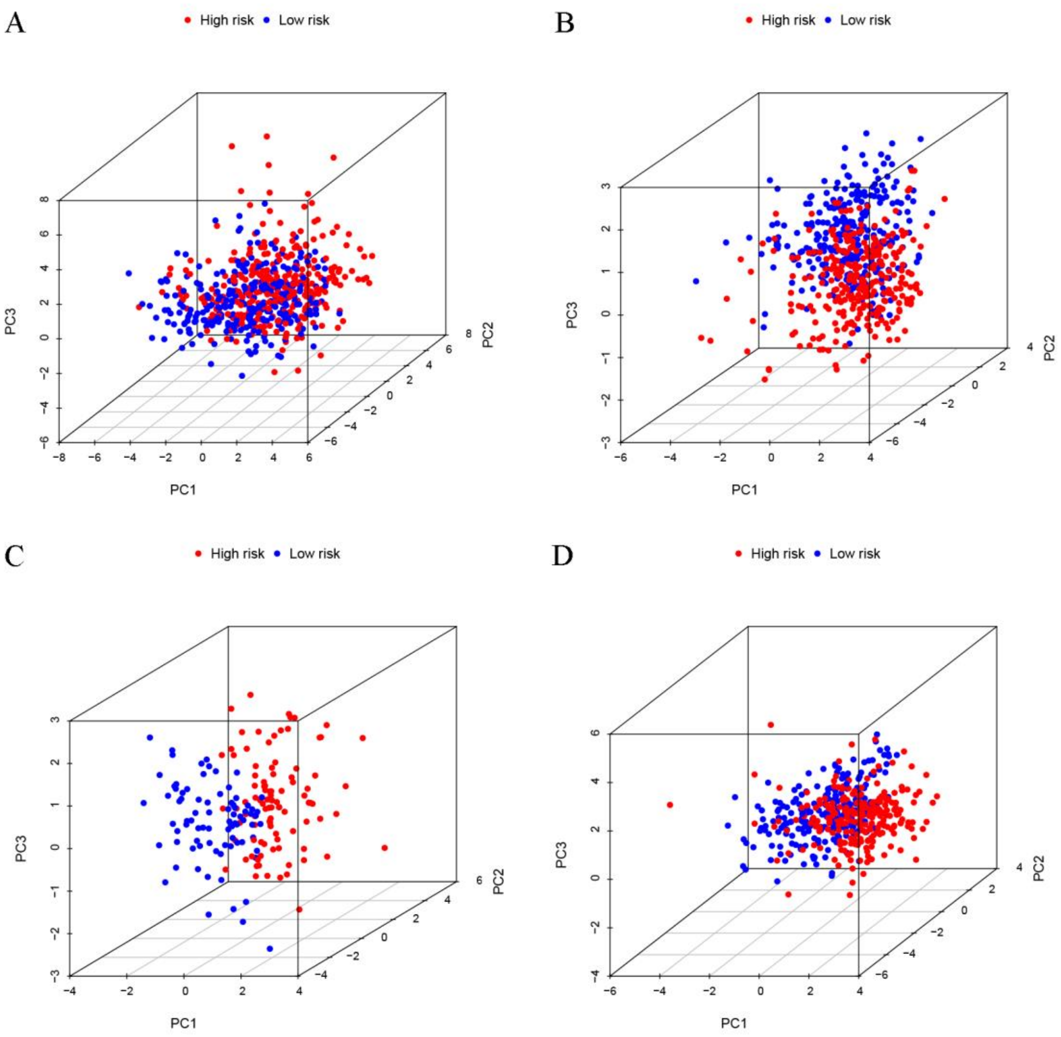
A
High risk
Low risk
PC1
PC2
PC3
B
High risk
Low risk
PC1
PC2
PC3
C
High risk
Low risk
PC1
PC2
PC3
D
High risk
Low risk
PC1
PC2
PC3

Figure S4

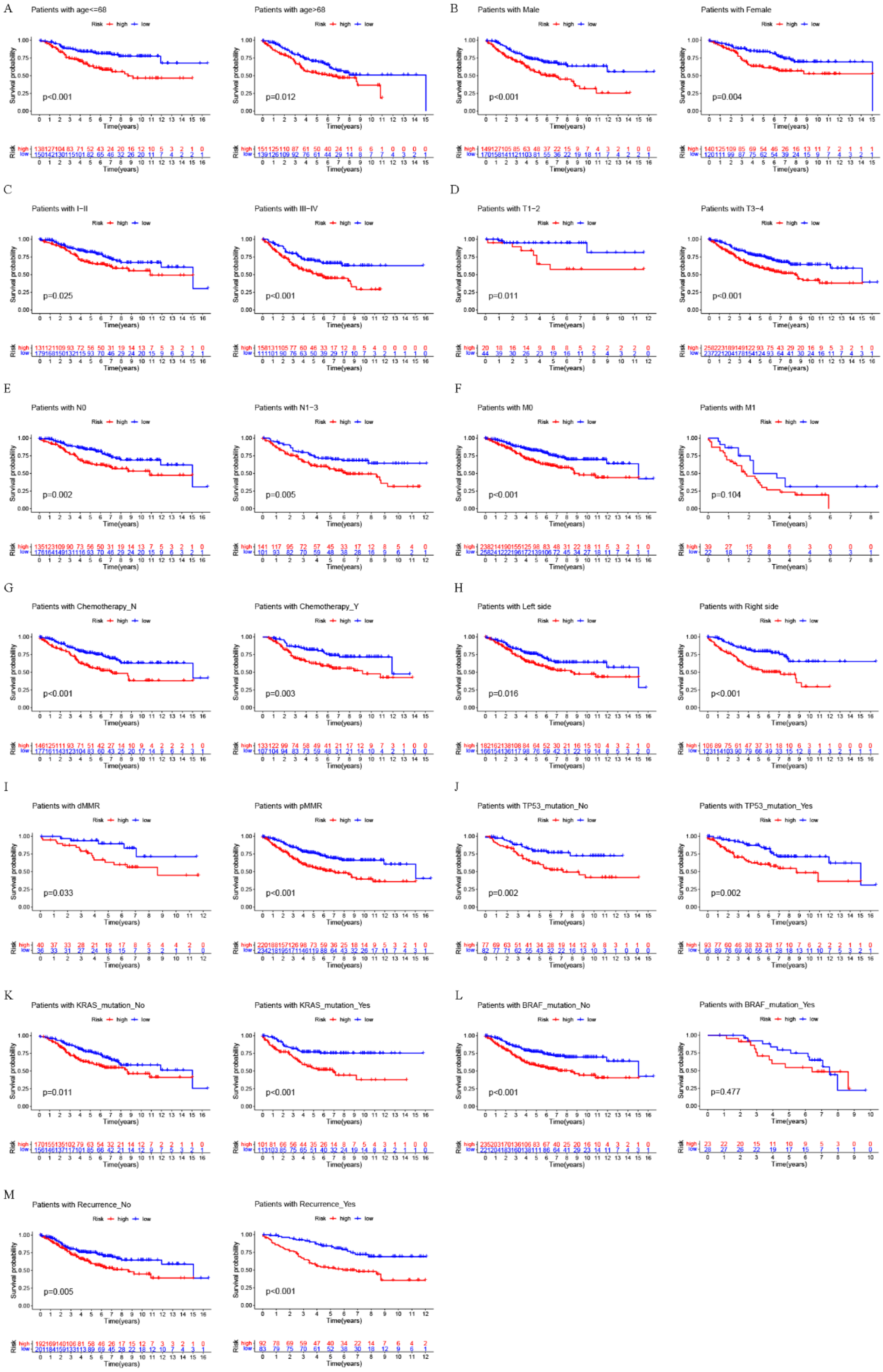
A
Patients with age<=68
p<0.001
Patients with age>68
p=0.012
B
Patients with Male
p<0.001
Patients with Female
p=0.004
C
Patients with I−II
p=0.025
Patients with III−IV
p<0.001
D
Patients with T1−2
p=0.011
Patients with T3−4
p<0.001
E
Patients with N0
p=0.002
Patients with N1−3
p=0.005
F
Patients with M0
p<0.001
Patients with M1
p=0.104
G
Patients with Chemotherapy_N
p<0.001
Patients with Chemotherapy_Y
p=0.003
H
Patients with Left side
p=0.016
Patients with Right side
p<0.001
I
Patients with dMMR
p=0.033
Patients with pMMR
p<0.001
J
Patients with TP53_mutation_No
p=0.002
Patients with TP53_mutation_Yes
p=0.002
K
Patients with KRAS_mutation_No
p=0.011
Patients with KRAS_mutation_Yes
p<0.001
L
Patients with BRAF_mutation_No
p<0.001
Patients with BRAF_mutation_Yes
p=0.477
M
Patients with Recurrence_No
p=0.005
Patients with Recurrence_Yes
p<0.001
Risk
high
low
Survival probability
Time(years)

Figure S5

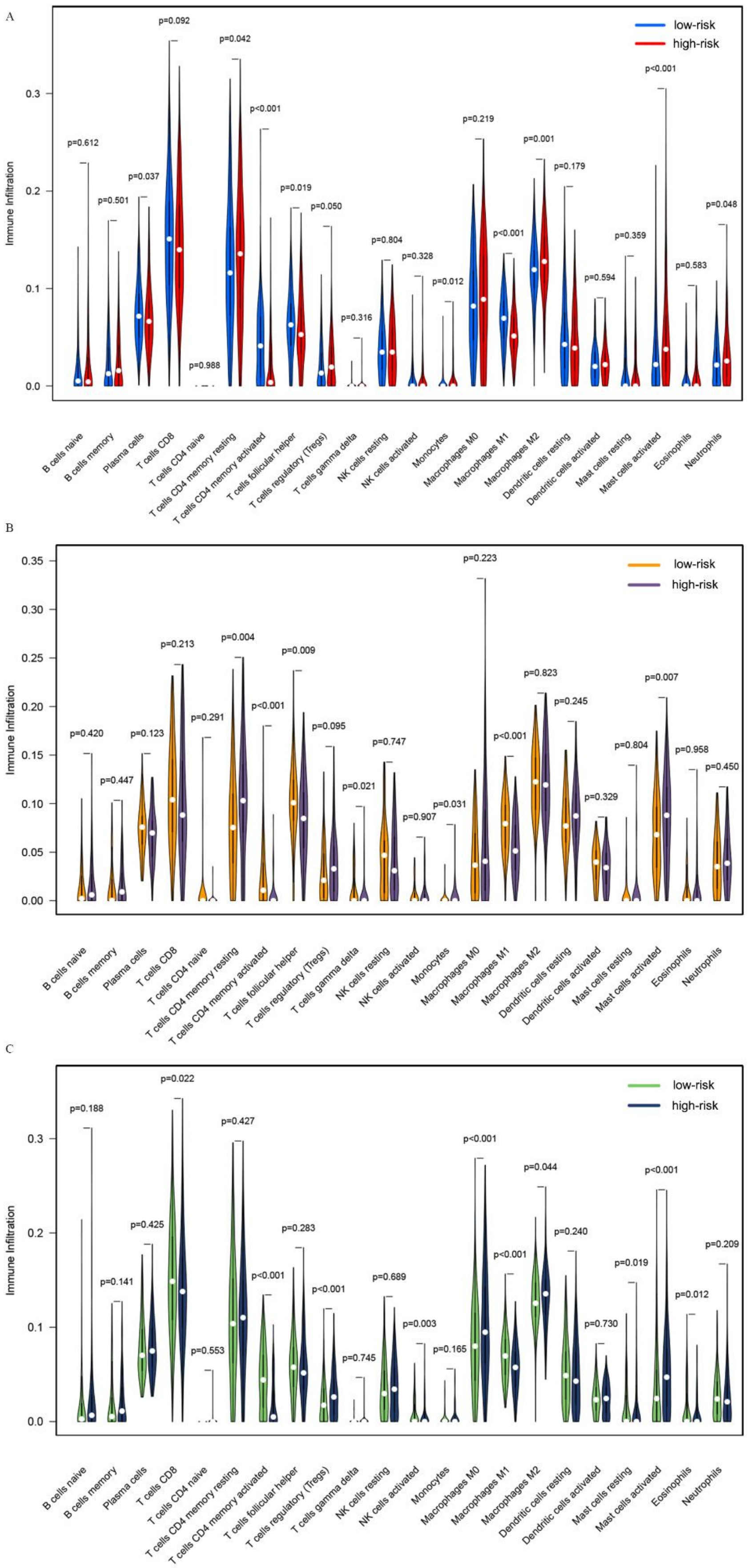
A
low-risk
high-risk
Immune Infiltration
0.0
0.1
0.2
0.3
p=0.612
p=0.501
p=0.037
p=0.092
p=0.988
p=0.042
p<0.001
p=0.019
p=0.050
p=0.316
p=0.804
p=0.328
p=0.012
p=0.219
p<0.001
p=0.001
p=0.179
p=0.594
p=0.359
p<0.001
p=0.583
p=0.048
B cells naive
B cells memory
Plasma cells
T cells CD8
T cells CD4 naive
T cells CD4 memory resting
T cells CD4 memory activated
T cells follicular helper
T cells regulatory (Tregs)
T cells gamma delta
NK cells resting
NK cells activated
Monocytes
Macrophages M0
Macrophages M1
Macrophages M2
Dendritic cells resting
Dendritic cells activated
Mast cells resting
Mast cells activated
Eosinophils
Neutrophils
B
low-risk
high-risk
Immune Infiltration
0.00
0.05
0.10
0.15
0.20
0.25
0.30
0.35
p=0.420
p=0.447
p=0.123
p=0.213
p=0.291
p=0.004
p<0.001
p=0.009
p=0.095
p=0.021
p=0.747
p=0.907
p=0.031
p=0.223
p<0.001
p=0.823
p=0.245
p=0.329
p=0.804
p=0.007
p=0.958
p=0.450
B cells naive
B cells memory
Plasma cells
T cells CD8
T cells CD4 naive
T cells CD4 memory resting
T cells CD4 memory activated
T cells follicular helper
T cells regulatory (Tregs)
T cells gamma delta
NK cells resting
NK cells activated
Monocytes
Macrophages M0
Macrophages M1
Macrophages M2
Dendritic cells resting
Dendritic cells activated
Mast cells resting
Mast cells activated
Eosinophils
Neutrophils
C
low-risk
high-risk
Immune Infiltration
0.0
0.1
0.2
0.3
p=0.188
p=0.141
p=0.425
p=0.022
p=0.553
p=0.427
p<0.001
p=0.283
p<0.001
p=0.745
p=0.689
p=0.003
p=0.165
p<0.001
p<0.001
p=0.044
p=0.240
p=0.730
p=0.019
p<0.001
p=0.012
p=0.209
B cells naive
B cells memory
Plasma cells
T cells CD8
T cells CD4 naive
T cells CD4 memory resting
T cells CD4 memory activated
T cells follicular helper
T cells regulatory (Tregs)
T cells gamma delta
NK cells resting
NK cells activated
Monocytes
Macrophages M0
Macrophages M1
Macrophages M2
Dendritic cells resting
Dendritic cells activated
Mast cells resting
Mast cells activated
Eosinophils
Neutrophils

Figure S6

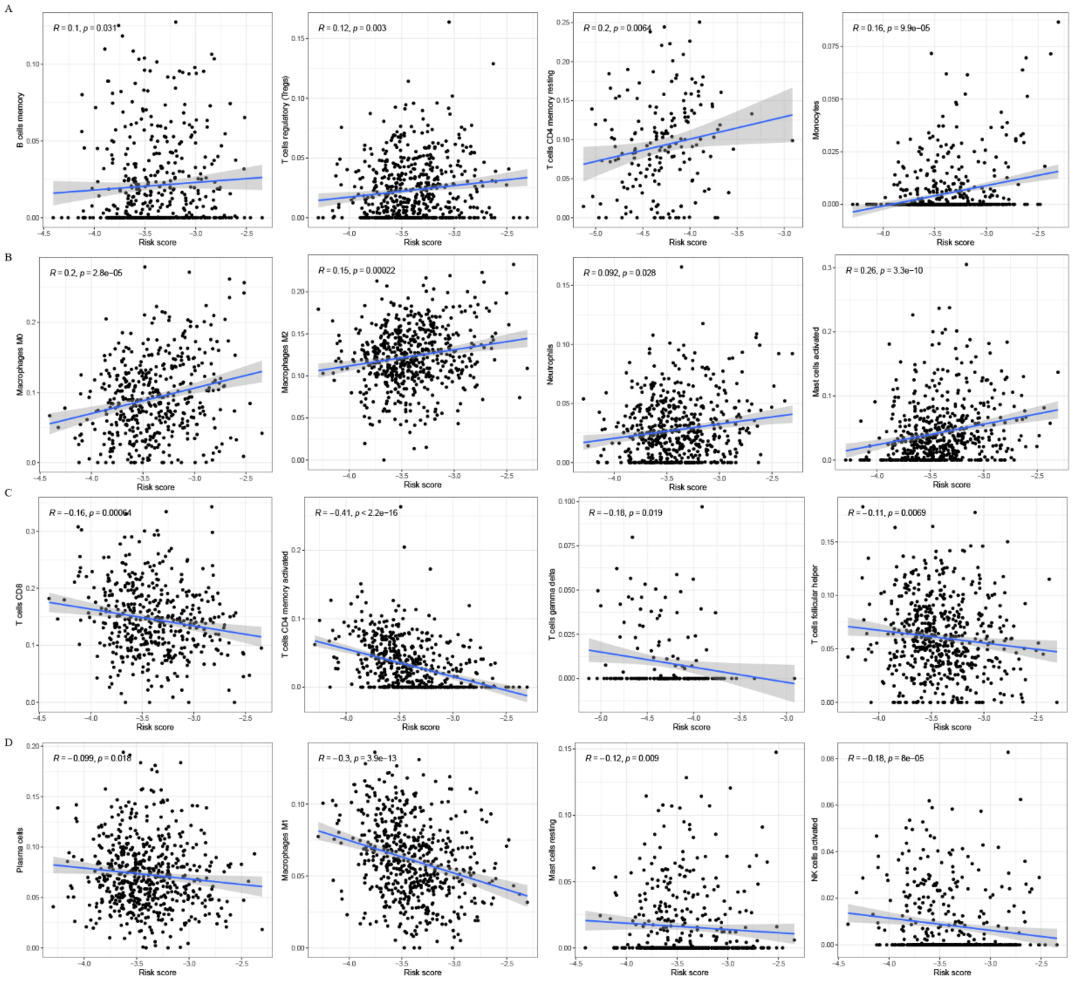

A
R = 0.1, p = 0.031
B cells memory
Risk score
R = 0.12, p = 0.003
T cells regulatory (Tregs)
Risk score
R = 0.2, p = 0.0064
T cells CD4 memory resting
Risk score
R = 0.16, p = 9.9e−05
Monocytes
Risk score
B
R = 0.2, p = 2.8e−05
Macrophages M0
Risk score
R = 0.15, p = 0.00022
Macrophages M2
Risk score
R = 0.092, p = 0.028
Neutrophils
Risk score
R = 0.26, p = 3.3e−10
Mast cells activated
Risk score
C
R = −0.16, p = 0.00064
T cells CD8
Risk score
R = −0.41, p < 2.2e−16
T cells CD4 memory activated
Risk score
R = −0.18, p = 0.019
T cells gamma delta
Risk score
R = −0.11, p = 0.0069
T cells follicular helper
Risk score
D
R = −0.099, p = 0.018
Plasma cells
Risk score
R = −0.3, p = 3.9e−13
Macrophages M1
Risk score
R = −0.12, p = 0.009
Mast cells resting
Risk score
R = −0.18, p = 8e−05
NK cells activated
Risk score

Figure S7

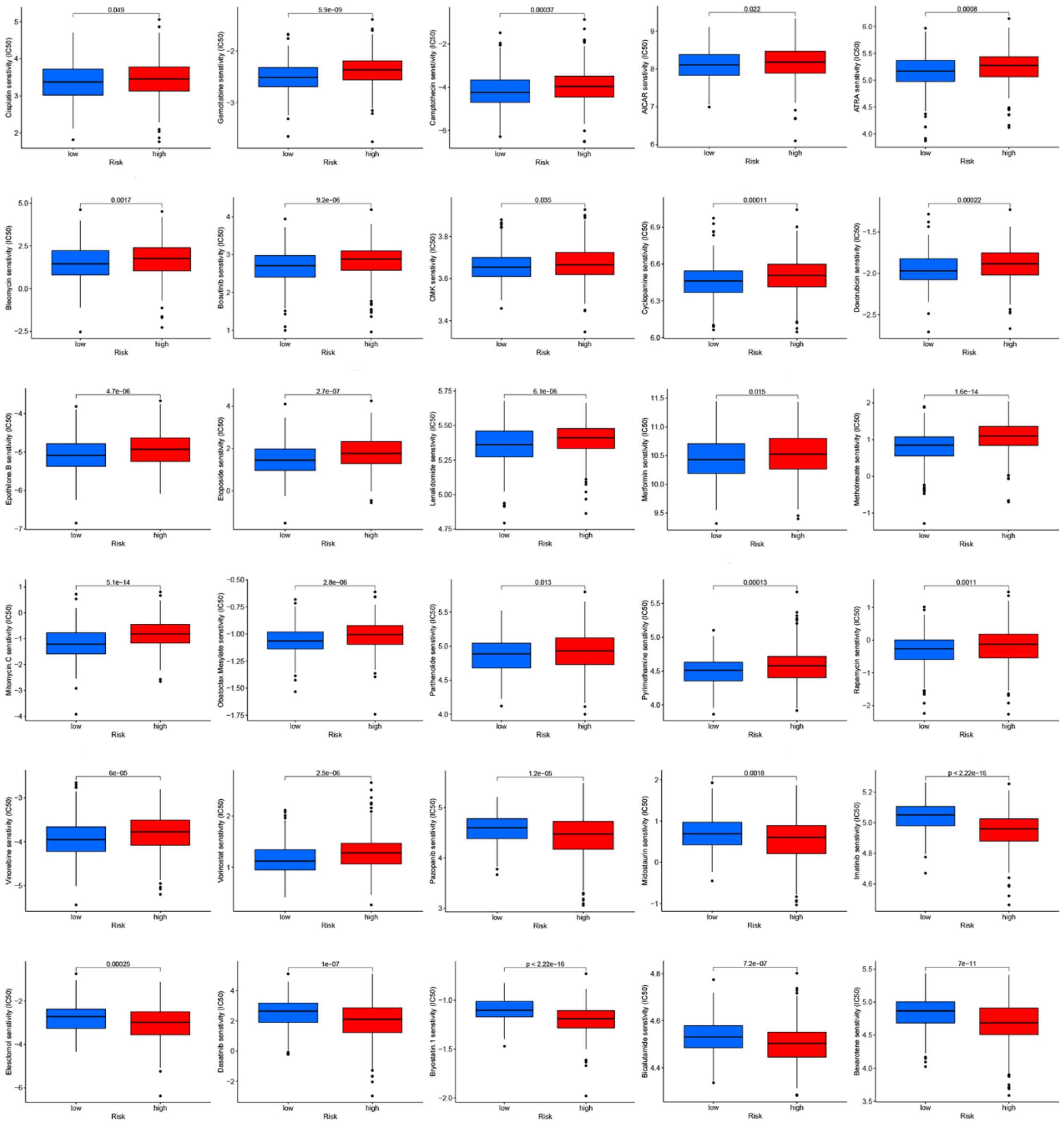

Cisplatin sensitivity (IC50)
0.049
Gemcitabine sensitivity (IC50)
5.9e-09
Camptothecin sensitivity (IC50)
0.00037
AICAR sensitivity (IC50)
0.022
ATRA sensitivity (IC50)
0.0008
Bleomycin sensitivity (IC50)
0.0017
Bosutinib sensitivity (IC50)
9.2e-06
CMK sensitivity (IC50)
0.035
Cyclopamine sensitivity (IC50)
0.00011
Doxorubicin sensitivity (IC50)
0.00022
Epothilone B sensitivity (IC50)
4.7e-06
Etoposide sensitivity (IC50)
2.7e-07
Lenalidomide sensitivity (IC50)
6.1e-06
Metformin sensitivity (IC50)
0.015
Methotrexate sensitivity (IC50)
1.6e-14
Mitomycin C sensitivity (IC50)
5.1e-14
Obatoclax Mesylate sensitivity (IC50)
2.8e-06
Parthenolide sensitivity (IC50)
0.013
Pyrimethamine sensitivity (IC50)
0.00013
Rapamycin sensitivity (IC50)
0.0011
Vinorelbine sensitivity (IC50)
6e-05
Vorinostat sensitivity (IC50)
2.5e-06
Pazopanib sensitivity (IC50)
1.2e-05
Midostaurin sensitivity (IC50)
0.0018
Imatinib sensitivity (IC50)
p < 2.22e-16
Elesclomol sensitivity (IC50)
0.00025
Dasatinib sensitivity (IC50)
1e-07
Bryostatin 1 sensitivity (IC50)
p < 2.22e-16
Bicalutamide sensitivity (IC50)
7.2e-07
Bexarotene sensitivity (IC50)
7e-11
low
high
Risk

Figure S8

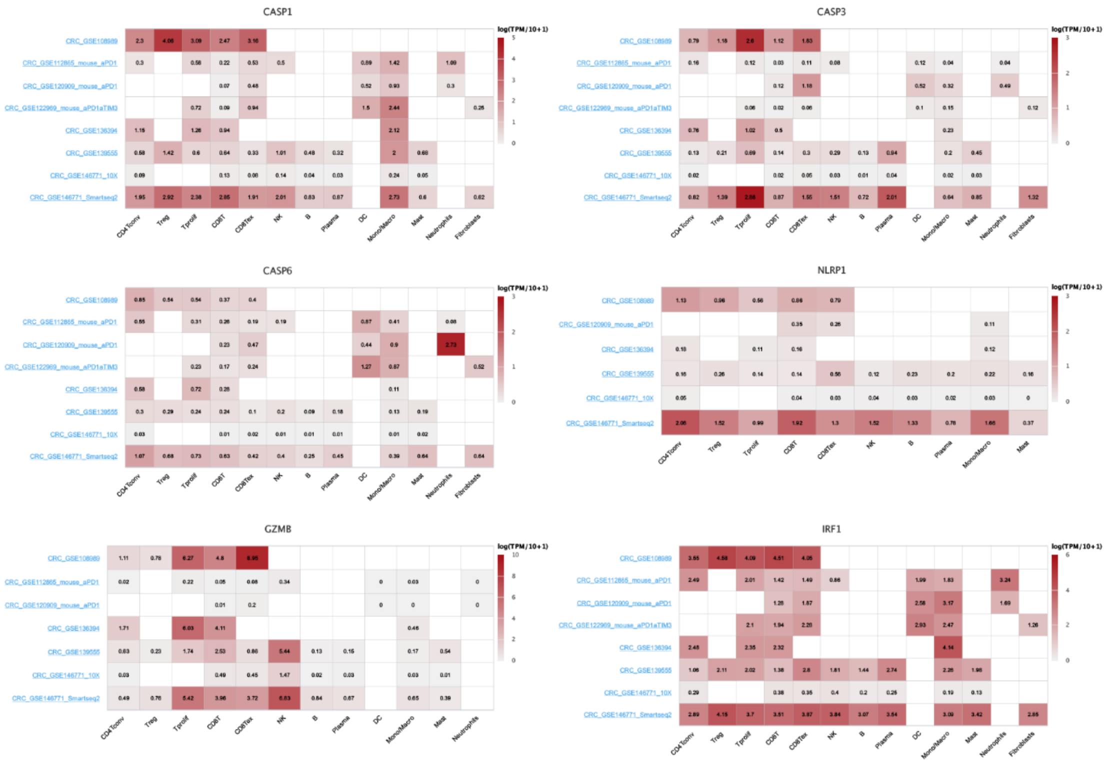

Figure S9

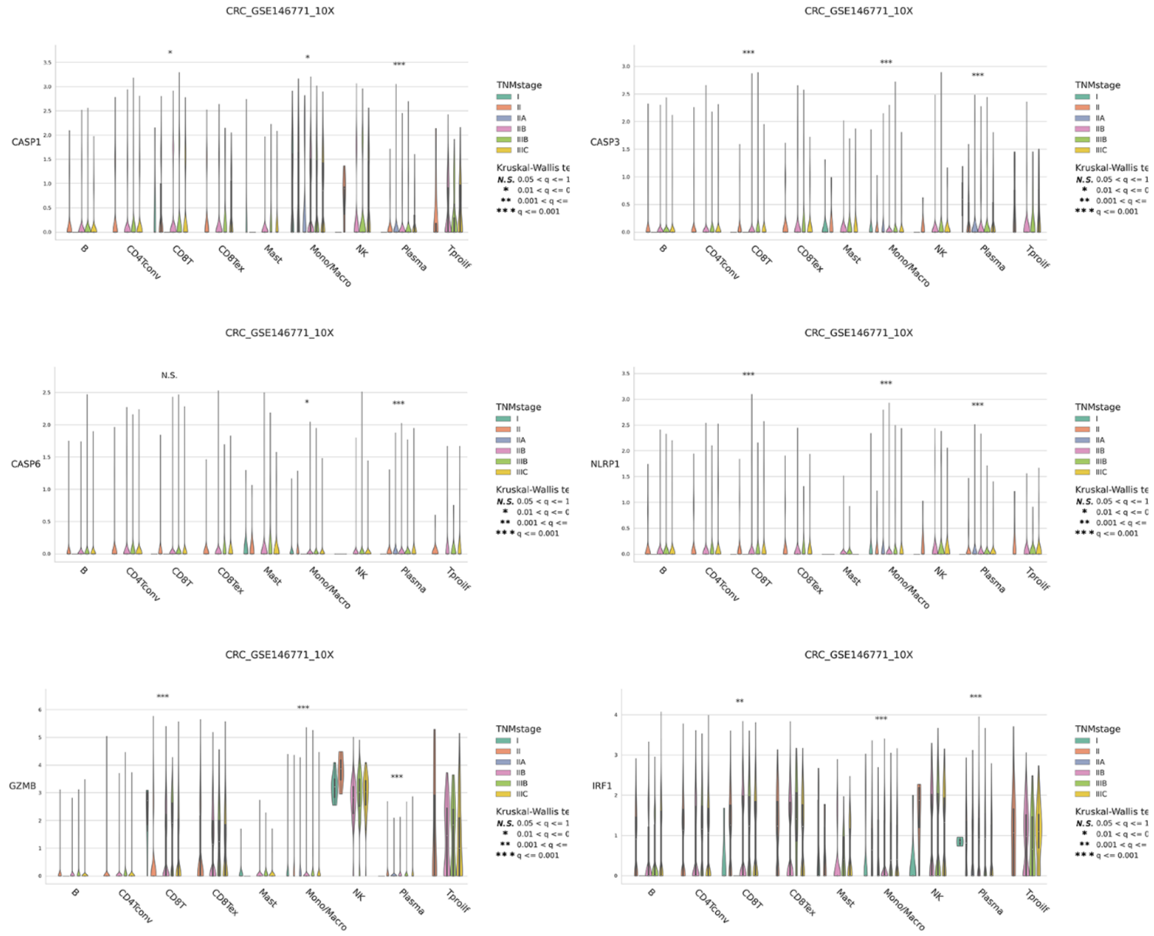

Figure S10

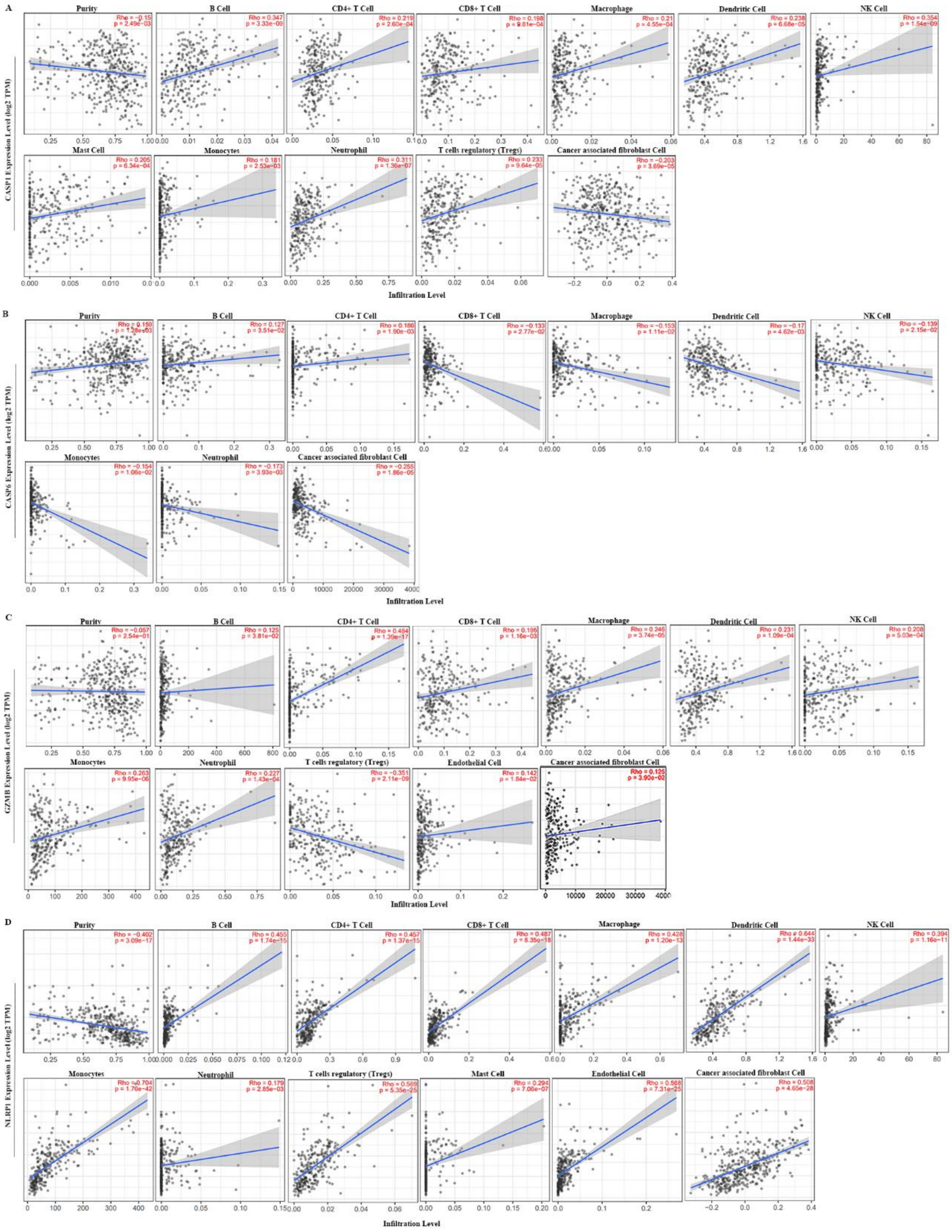
A
Purity
B Cell
CD4+ T Cell
CD8+ T Cell
Macrophage
Dendritic Cell
NK Cell
Mast Cell
Monocytes
Neutrophil
T cells regulatory (Tregs)
Cancer associated fibroblast Cell
CASP1 Expression Level (log2 TPM)
Infiltration Level
B
Purity
B Cell
CD4+ T Cell
CD8+ T Cell
Macrophage
Dendritic Cell
NK Cell
Monocytes
Neutrophil
Cancer associated fibroblast Cell
CASP6 Expression Level (log2 TPM)
Infiltration Level
C
Purity
B Cell
CD4+ T Cell
CD8+ T Cell
Macrophage
Dendritic Cell
NK Cell
Monocytes
Neutrophil
T cells regulatory (Tregs)
Endothelial Cell
Cancer associated fibroblast Cell
GZMB Expression Level (log2 TPM)
Infiltration Level
D
Purity
B Cell
CD4+ T Cell
CD8+ T Cell
Macrophage
Dendritic Cell
NK Cell
Monocytes
Neutrophil
T cells regulatory (Tregs)
Mast Cell
Endothelial Cell
Cancer associated fibroblast Cell
NLRP1 Expression Level (log2 TPM)
Infiltration Level

Figure S11

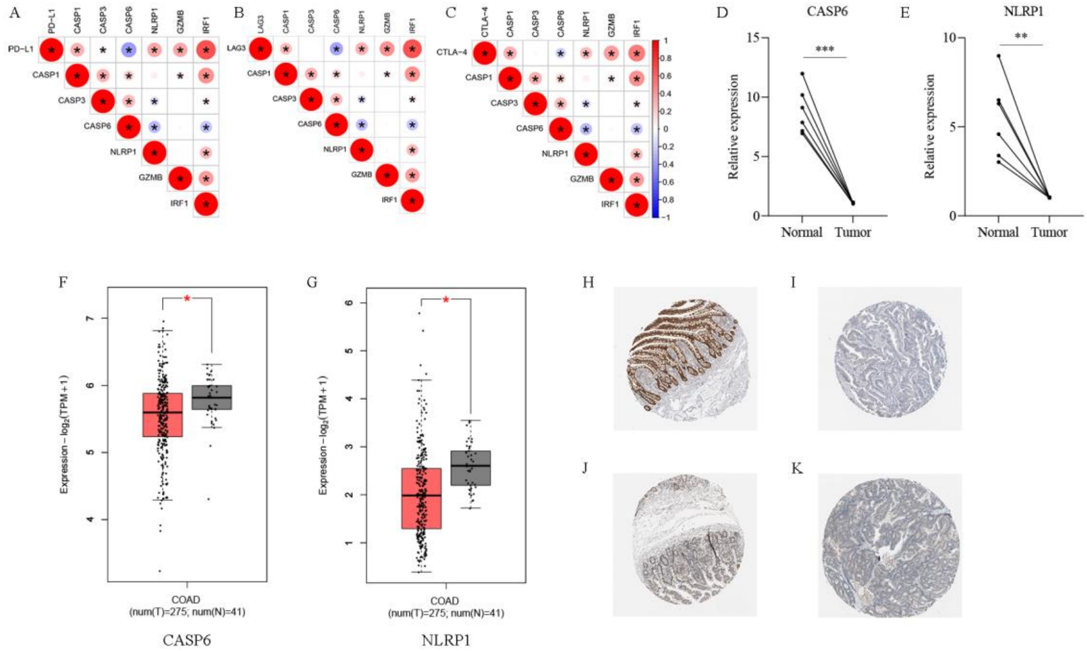

Figure S12

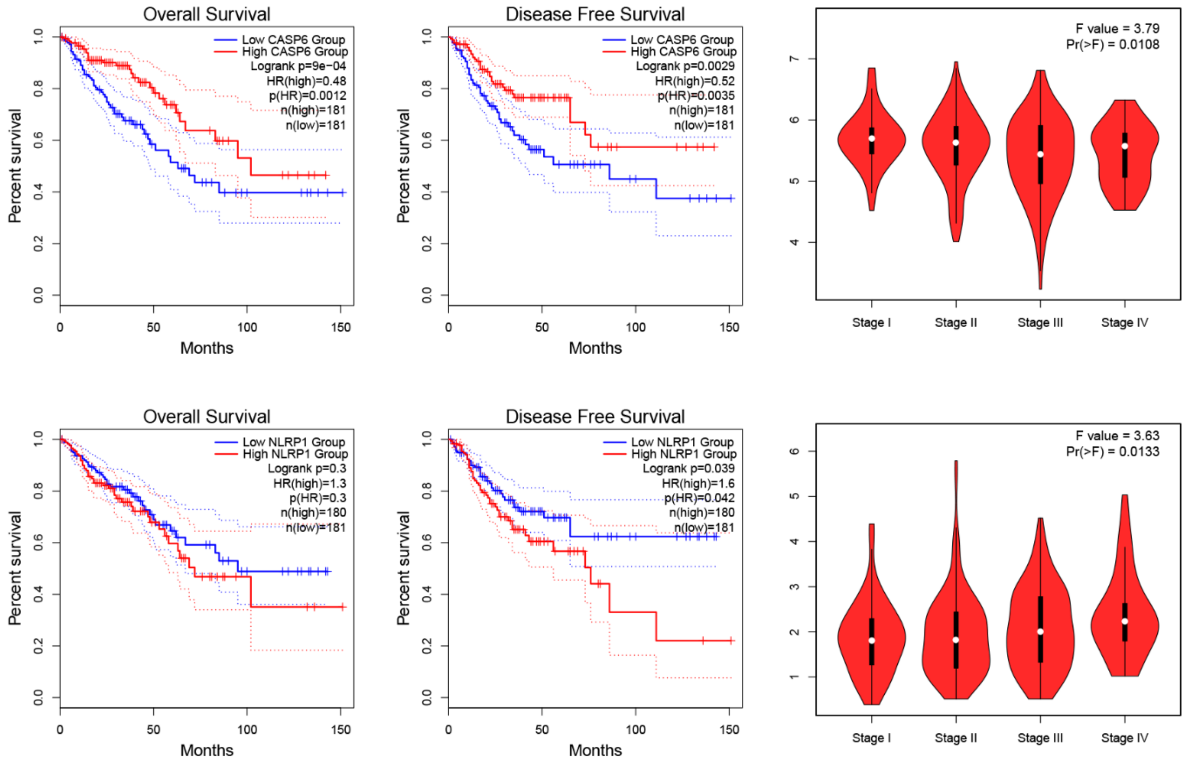

Figure S13

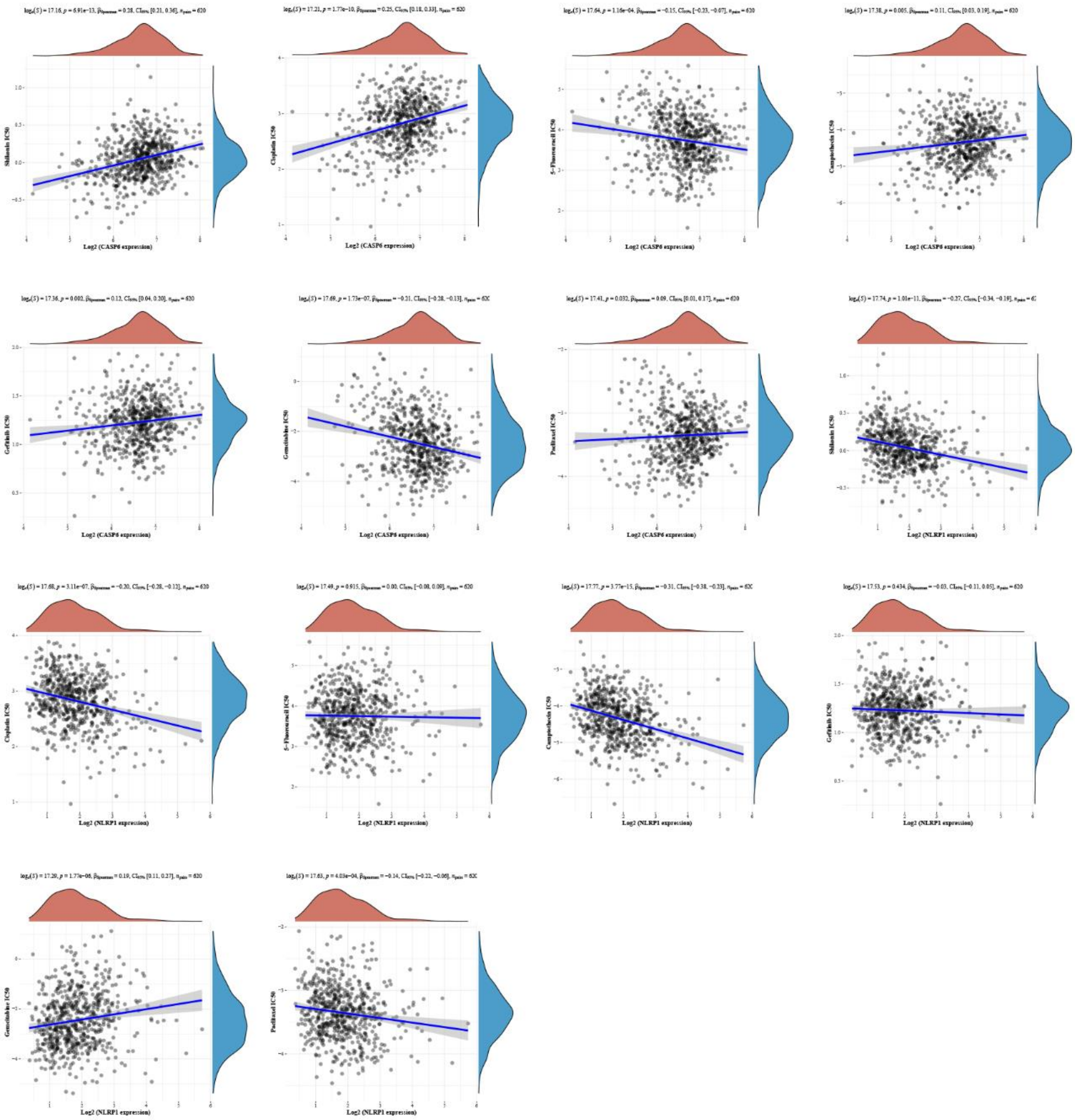

Figure S14

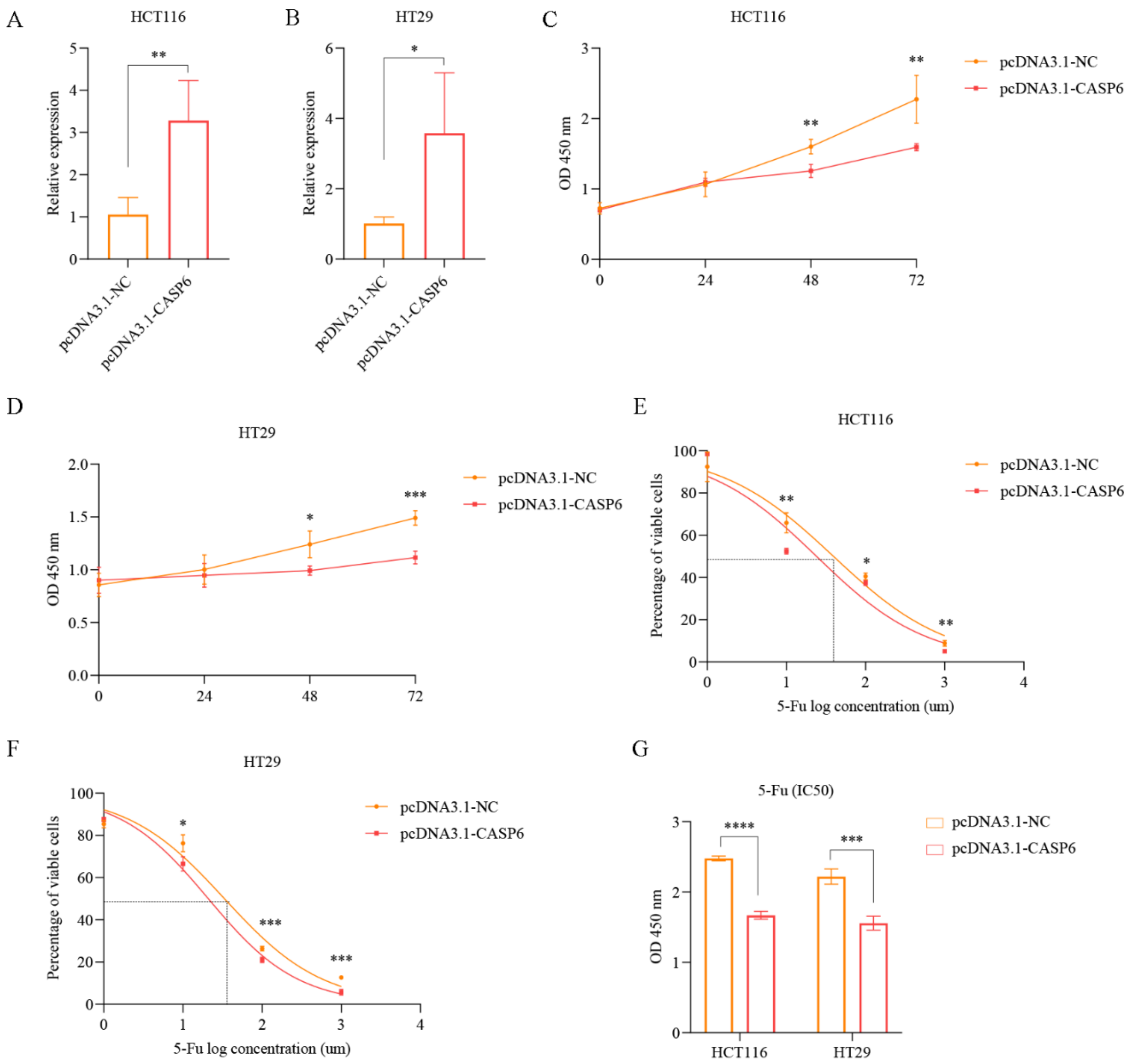

A
HCT116
Relative expression
pcDNA3.1-NC
pcDNA3.1-CASP6
B
HT29
Relative expression
pcDNA3.1-NC
pcDNA3.1-CASP6
C
HCT116
OD 450 nm
pcDNA3.1-NC
pcDNA3.1-CASP6
D
HT29
OD 450 nm
pcDNA3.1-NC
pcDNA3.1-CASP6
E
HCT116
Percentage of viable cells
5-Fu log concentration (um)
pcDNA3.1-NC
pcDNA3.1-CASP6
F
HT29
Percentage of viable cells
5-Fu log concentration (um)
pcDNA3.1-NC
pcDNA3.1-CASP6
G
5-Fu (IC50)
OD 450 nm
HCT116
HT29
pcDNA3.1-NC
pcDNA3.1-CASP6

Figure S15
