# Supplementary material for: Pyroptosis-Related Signature as Potential Biomarkers for Predicting Prognosis and Therapy Response in Colorectal Cancer Patients
Source: Front Genet. 2022 Jul 22;13:925338. doi: 10.3389/fgene.2022.925338 (PMC9355164; doi:10.3389/fgene.2022.925338)
Supplement: Supplementary file 5 [file Table4.DOCX]

Table S7. The differentially expressed miRNAs binding with CASP6 in CRC

| Binding Gene | Mirnaid | Binding P-value | Accessibility | Number of Pairings | Binding Region Length | Longest Consecutive Pairings |
| --- | --- | --- | --- | --- | --- | --- |
| CASP6 | hsa-miR-509-3-5p | 1 | 1.45*10^-5^ | 16 | 21 | 5 |
|  | hsa-miR-3122 | 1 | 1.58*10^-5^ | 11 | 16 | 7 |
|  | hsa-miR-4494 | 1 | 0.001194613 | 17 | 24 | 12 |
|  | hsa-miR-4763-3p | 1 | 0.000204951 | 17 | 26 | 7 |
|  | hsa-miR-6086 | 1 | 3.8*10^-5^ | 17 | 29 | 11 |
|  | hsa-miR-340-3p | 1 | 0.000497733 | 18 | 25 | 9 |
|  | hsa-miR-4425 | 1 | 1.17*10^-5^ | 15 | 16 | 15 |
|  | hsa-miR-6075 | 1 | 0.000204951 | 17 | 25 | 8 |
